# Sulindac exhibits anti-proliferative and anti-invasive effects and enhances the sensitivity to paclitaxel in ovarian cancer

**DOI:** 10.3389/fphar.2025.1520771

**Published:** 2025-04-30

**Authors:** Shuning Chen, Weimin Kong, Xiaochang Shen, Nikita Sinha, Jennifer Haag, Boer Deng, Haomeng Zhang, Catherine John, Wenchuan Sun, Chunxiao Zhou, Victoria L. Bae-Jump

**Affiliations:** ^1^ Department of Gynecology, Beijing Obstetrics and Gynecology Hospital, Beijing Maternal and Child Healthcare Hospital, Capital Medical University, Beijing, China; ^2^ Division of Gynecologic Oncology, Department of Obstetrics and Gynecology, University of North Carolina at Chapel Hill, Chapel Hill, NC, United States; ^3^ Lineberger Comprehensive Cancer Center, University of North Carolina at Chapel Hill, Chapel Hill, NC, United States

**Keywords:** ovarian cancer, sulindac, cell proliferation, invasion, synergy, paclitaxel

## Abstract

**Objective:**

Chronic inflammation is a key contributor to carcinogenesis, progression, and chemoresistance in ovarian cancer, making inflammatory pathways a logical therapeutic target for the treatment of this disease. Sulindac, a commonly used non-steroidal anti-inflammatory drug, has demonstrated anti-proliferative and anti-invasive effects on several preclinical models of cancer. In this study, we investigated the antitumorigenic effects of sulindac in human ovarian cancer cell lines and a transgenic mouse model of ovarian cancer (KpB).

**Methods:**

MTT and colony formation assays were used to evaluate cell proliferation. Cell cycle was detected by Cellometer. ELISA assays were conducted to evaluate the changes of cellular stress, apoptosis and adhesion, while invasion was determined by wound healing assay. Protein expression was examined through Western blotting and immunohistochemistry.

**Results:**

Our results demonstrated that sulindac significantly inhibited cell proliferation, induced cellular stress and apoptosis, caused G1 phase cell cycle arrest, and reduced cell invasion, and suppressed Cox-2 and NF-κB pathways in the MES and OVCAR5 cell lines. Inhibition of cellular stress by N-acetylcysteine partially reversed the anti-proliferative and anti-invasive effects of sulindac. The combination of sulindac and paclitaxel produced synergistic effects in inhibiting cell growth in both paclitaxel sensitive and resistant MES cells. Treatment with sulindac for 4 weeks effectively reduced tumor growth, improved serum levels of inflammatory cytokines and chemokines, and reduced the expression of Cox-2 of ovarian tumors in KpB mice compared with untreated mice.

**Conclusions:**

These findings provide support for the development of clinical trials repurposing sulindac in the treatment of OC, possibly in combination with paclitaxel.

## Introduction

Ovarian cancer (OC) is the deadliest gynecologic malignancy and ranks as the 6th leading cause of cancer-related mortality among women in the United States (Siegel et al., 2024). Inflammation, obesity, reproductive history, age, hormone replacement, endometriosis and genetic predispositions (such as BRCA1/2 and p53 mutations) are known risk factors for OC (Webb and Jordan, 2024). Due to its asymptomatic nature in the early stage and lack of effective population-based screening, approximately 75% of OC patients are diagnosed at an advanced stage (Lheureux et al., 2019; Kuroki and Guntupalli, 2020). The standard treatment regimen for OC patients is tumor debulking surgery followed by adjuvant systemic therapy, primarily the combination chemotherapy of paclitaxel (PTX) and carboplatin. Even though many patients achieve remission with initial therapy, the 5-year survival rate is only 50% because of chemoresistance and recurrence rates as high as 80% (Kuroki and Guntupalli, 2020; Gupta et al., 2019). Thus, innovative therapeutic strategies are urgently needed to improve prognosis in advanced and relapsed OC patients.

Chronic inflammation caused by ovulation, pelvic inflammatory disease, endometriosis and obesity has been linked to increased risk of OC (Aceto and Cheon, 2023). Numerous inflammatory cytokines and chemokines, including tumor necrosis factor-alpha (TNF-α), interleukin-1 beta (IL-1β), interleukin-6 (IL-6), prostaglandin E2 (PGE2) and cyclooxygenase-2 (Cox-2), promote cell proliferation, malignant transformation, and genetic and epigenetic alternations in the development of OC through diverse inflammatory pathways (Aggarwal et al., 2006; Neuhaus et al., 2024). There is growing evidence that long-term use of nonsteroidal anti-inflammatory drugs (NSAIDs) may reduce the risk of certain types of cancer, such as colorectal cancer and OC (Hurwitz et al., 2022; Heer et al., 2022; Micha et al., 2024; Zheng et al., 2024). A recent meta-analysis of 17 large-scale study populations demonstrated that frequent aspirin use (6 days/week) was associated with a 13% lower risk of OC in women with at least one risk factor, indicating that NSAIDs have anti-tumorigenic potential in OC (Hurwitz et al., 2022). Sulindac is a widely used clinical NSAID that successfully treats inflammatory diseases by inhibiting the Cox-2 signaling pathway. Multiple epidemiological studies have shown that sulindac has a positive chemopreventive effect on colorectal cancer by causing regression of colorectal adenomatous polyps (Heer et al., 2022). Preclinical studies have also found that sulindac exerts anti-proliferative and anti-invasive activities effects on different types of cancer *in vitro* and *in vivo* through Cox-2-dependent or -independent pathways (Giardiello et al., 1993; Madka et al., 2023; Waddell and Loughry, 1983; Kim et al., 2015; Mladenova et al., 2013; Huang et al., 2001; Poursoltani et al., 2021), and synergistically increases the sensitivity of paclitaxel, tamoxifen, and EGFR inhibitors in breast cancer, lung cancer, and uterine fibroids (Soriano et al., 1999; Hansmann et al., 2004; Samadder et al., 2018; Hossain et al., 2023).

Overexpression of Cox-2 is significantly associated with advanced stage, high tumor grade, and reduced disease-free survival and overall survival (OS) in patients with OC (Li et al., 2004; Seo et al., 2004; Ferrandina et al., 2002; Sun et al., 2017). Increased Cox-2 activity promotes cell proliferation, reduces apoptosis, enhances invasive capacity, and increases chemoresistance in OC cells (Gu et al., 2008; Deng et al., 2020; Jin et al., 2025; Lulli et al., 2024). Targeting Cox-2 by siRNA or small molecule inhibitors effectively inhibits cell viability, migration and invasion, tumor growth, and reverses chemoresistance in preclinical models of OC (Gu et al., 2008; Deng et al., 2020). Our earlier work showed celecoxib, a small molecule inhibitor of Cox-2, exhibited anti-proliferative and anti-invasive effects in OC cell lines and an animal model of OC (Suri et al., 2016). These results indicate that inhibition of the inflammatory response through targeting Cox-2 may be a good strategy for the treatment of OC. Therefore, this study aimed to investigate the anti-tumorigenic effects of sulindac and the combination of sulindac and paclitaxel in human OC cell lines and a transgenic mouse model of OC, with the goal of providing a novel approach to addressing the pressing needs for more effective treatments for patients with OC.

## Materials and methods

### Cell culture and reagents

The human ovarian cancer cell lines OV433, OVCAR5, OVCAR3, MES, and paclitaxel-resistant MES-TP were utilized in this study. The MES-TP cell line was kindly provided by Dr. Sikic (Stanford University School of Medicine) and was established by stepwise exposure of MES cells to increasing concentrations of paclitaxel in combination with the P-glycoprotein inhibitor PSC833 (Moisan et al., 2014). This cell line is at least 10-fold more resistant to paclitaxel than its parental cell line (MES), with increased expression of P-glycoprotein (Zhang et al., 2023). Both MES and MES-TP were grown in McCoy’s 5A medium. OV433 cells were cultured in the RPMI 1640 medium, while OVCAR5 and OVCAR3 cells were maintained in the DMEM/F12 medium. All mediums contained 10% fetal bovine serum (FBS) (Thermo Fisher Scientific; Waltham, MA), 1% penicillin/streptomycin, and 1% L-Glutamine (Gibco Cell Culture, CA). All cell lines were cultured under humidified conditions with 5% CO_2_ at 37°C. Sulindac sulfide was purchased from MedChemExpress (Newark, NJ). MTT, N-acetylcysteine (NAC), Z-VAD-FMK, and paclitaxel (PTX) were purchased from Sigma-Aldrich (St. Louis, MO). Antibodies used were from Cell Signaling Technology (Beverly, MA) and ABclonal (Woburn, MA). The Western Lightning™ Plus Chemiluminescence Reagent was purchased from PerkinElmer (Waltham, MA).

### MTT assay

4,000–8,000 cells per well were incubated in 96-well plates overnight and treated with various doses of sulindac (ranging from 10 to 500 μM) for 72 h 5 μL of MTT solution (5 mg/mL) was added to each well, and the plate was incubated for another 1 h at 37°C. The absorbance of MTT was measured at 562 nm using a microplate reader (Tecan; Morrisville, NC) after adding 100 μL DMSO to each well. The effect of sulindac on OC cell proliferation was evaluated as a percentage of control cell growth, and then IC50 analysis was determined using the AAT Bioquest calculator (Sunnyvale, CA).

### Colony formation assay

The MES cells and OVCAR5 cell lines were plated at a density of 200 and 400 cells/well in 6-well plates respectively, overnight. Subsequently, the plates were treated with varying concentrations of sulindac (25, 75, and 100 μM) for 72 h. The cells were cultured continuously for 14 days, and the medium was changed every 3 days. Cells were stained with 0.5% crystal violet, and colonies were counted under a microscope for analysis.

### Reactive oxygen species (ROS) assay

Intracellular ROS levels were quantified by DCFH-DA assay. The MES and OVCAR5 cells were seeded at a density of 8,000 cells/well in 96-well plates overnight, and then treated with 25, 75, and 100 μM sulindac for 8 h at 37°C. After treatment, the supernatants were removed, and phenol red-free medium containing 15 µM DCFH-CA (Sigma-Aldrich) was added to each well for an additional 30-minute incubation. The fluorescence intensity was then measured at Ex/Em 485/525 nm using a Tecan microplate reader.

### JC-1 assay

The MES and OVCAR5 cells were cultured at 1.5 × 10^4^ cells/well and 2.5 × 10^4^ cells/well, respectively, in 96-well plates overnight. The cells were treated with sulindac at doses of 25, 75, and 100 μM for 6 h and then incubated with 200 µM JC-1 dye (Sigma-Aldrich) for 30 min at 37°C in the dark. After washing each well with warm PBS, the green JC-1 signal was detected at Ex/Em 485/535 nm, and the red JC-1 signal was measured at Ex/Em 535/590 nm using a Tecan microplate reader.

### TMRE assay

The MES and OVCAR5 cells, plated at 1 × 10^5^ and 1.5 × 10^5^ cells/well, respectively, were incubated in black clear-bottom 96-well plates overnight. The cells were then exposed to 25, 75, and 100 μM sulindac for 6 h. After removing the supernatants, 100 µL of 1 mM TMRE (Sigma-Aldrich) was added to each well and incubated for an additional 30 min at 37°C in the dark. After washing each well with PBS, the plate was measured at Ex/Em 549/575 nm using a Tecan microplate reader.

### Cell cycle analysis

The MES and OVCAR5 cells were plated in 6-well plates at a density of 2.5 × 10^5^ cells overnight, and then treated with 25, 75, and 100 µM sulindac for 24 h. After treatment, the cells were harvested using 0.25% trypsin (Thermo Fisher Scientific) and fixed in 100% methanol for 30 min. The cells were then resuspended in a solution containing 10 mM propidium iodide (PI), 50 ug RNase A, and 0.05% Triton X-100 and then incubated at 37°C for 30 min in the dark. Cell cycle progression was measured by Cellometer (Nexcelom, Lawrence, MA), and the results were analyzed using FCS7 Express software (Molecular Devices, Sunnyvale, CA).

### Cleaved caspase 3, 8, and 9 ELISA assays

The MES and OVCAR5 cells were cultured at a concentration of 2.5 × 10^5^ cells/mL in 6-well plates overnight, followed by treatment with sulindac at 25, 75, and 100 μM for 14 h. The cells were then lysed using 150 µL/well of 1X caspase lysis buffer, and the protein concentration was determined using the BCA assay (Thermo Fisher Scientific). Lysates (30–40 µg) were added to a black clear bottom 96-well plate and incubated with reaction buffer containing 200 µM caspase substrates for 30 min. Specifically, the substrates Ac-DEVD-AMC, Ac-IETD-AFC, and Ac-LEHD-AMC (AAT Bioquest) were used to detect cleaved caspase 3, 8, and 9 activities, respectively. The fluorescence intensity of cleaved caspase 3 and 9 was measured at an excitation/emission (Ex/Em) wavelengths of 341/441 nm, while the fluorescence intensity of cleaved caspase 8 was measured at Ex/Em 376/482 nm using a Tecan microplate reader.

### Adhesion assay

96-well plates were coated with 80 μL of laminin-1 (10 μg/mL) (Sigma-Aldrich) and incubated overnight at 4°C before use. After each well was treated with a blocking buffer (0.5% BSA in PBS) for 30 min, 1 × 10^5^/well of MES and OVACR5 cells, and varying concentration of sulindac (25, 75, and 100 μM) were added, followed by incubation for 90 min. After aspirating the supernatants, the adhered cells were fixed with 5% glutaraldehyde for 30 min and stained with 0.1% crystal violet for 15 min at room temperature. After washing twice with PBS, 100 µL of 10% acetic acid was added to each well to dissolve the dye. The absorbance was read at 575 nm using a Tecan microplate reader.

### Wound healing assay

The MES and OVCAR5 cells were seeded in 6-well plates at a density of 5 × 10^5^/well and cultured overnight. After replacing the culture medium to containing 1% FBS, three uniform wounds were scratched across the cell monolayer using a 200 μL pipette tip. The cells were then gently washed twice with PBS and treated with sulindac (5, 10, and 25 μM) for 24 h. Images of each well were taken using a microscope at 24 h after treatment. Wound width was measured and calculated using the ImageJ software (National Institutes of Health; Bethesda, MD).

### Western immunoblotting

The MES and OVCAR5 cells were seeded at a density of 2.5 × 10^5^ cells/well in 6-well plates and cultured overnight. Then cells were treated with sulindac at doses of 25, 75, and 100 μM for 8–24h. Total cell lysate was prepared using pre-cold RIPA lysis buffer, and the protein concentration was determined by BCA assay (Thermo Fisher Scientific). Equal amounts of 20–25 µg protein were electrophoresed on 10% or 12% SDS-PAGE and transferred onto PVDF membranes (Bio-Rad; Hercules, CA) at 4°C for 90–120 min. After blocking with 5% fat-free milk for 1 h at room temperature, the membranes were incubated overnight at 4°C with appropriate primary antibodies. The detailed information of primary antibodies was listed in (Supplementary Table S1). The next day, membranes were washed with TBS-T buffer and then incubated with secondary antibodies for 1 h at room temperature. Membranes were developed using the Western Lightning™ Plus-ECL (PerkinElmer) and visualized using the ChemiDoc Image System (Bio-Rad, Hercules, CA). The quantitative assessment of protein expression was analyzed by ImageJ software.

### KpB transgenic mouse model of OC

The K18−gT_121_^+/−^; p53^fl/fl^; Brca1^fl/fl^ (KpB) mouse model of high-grade serous epithelial OC has been described previously (Makowski et al., 2014; Szabova et al., 2012). Our animal protocol (21–229) was approved by the Institutional Animal Care and Use Committee (IACUC) at the University of North Carolina at Chapel Hill. For the induction of ovarian tumors, 5 ul of 2.5 × 10^7^ P.F.U of recombinant adenovirus Ad5-CMV-Cre (AdCre, Transfer Vector Core, University of Iowa) was injected into the left ovarian bursa cavity of KpB mice at 6–8 weeks of age. Mice were examined weekly by abdominal palpation for the appearance of ovarian tumors. When ovarian tumors reached 0.1–0.2 cm in diameter, the mice were randomly divided into two groups (15 mice per group): a control group and a sulindac-treated group. Mice in the treatment group were treated with 7.5 mg/kg of sulindac daily via oral gavage for 4 weeks. Throughout the treatment period, all animals were checked daily for any signs of toxicity and weighed weekly. The size of ovarian tumors was checked twice a week by palpation. Mice were euthanized by CO_2_ asphyxiation after finishing the treatment. Ovarian tumors and blood samples were weighted and stored at −80°C until further use. Ovarian tumor volumes were calculated using the formula: (width^2^ × length)/2.

### Immunohistochemistry (IHC) of ovarian tumors

The mouse ovarian tumor tissues were fixed in formalin, then paraffin-embedded and sectioned into 4 μm slides at the Animal Histopathology Core Facility at UNC-CH. The slides were deparaffinized with xylene and hydrated with ethanol. Subsequently, fresh antigen retrieval buffer was used to boil the slides for 3 min in a pressure cooker, followed by soaking in cold water for 10 min. To block nonspecific binding, the slides were treated with a protein block solution (Dako, Agilent Technologies, Santa Clara, CA) for 1 h at room temperature and then incubated with primary antibodies for Ki-67 (1:400), Bcl-xL (1: 1,200), and Cox-2 (1:200) overnight at 4°C. Slides were washed three times with TBST buffer and then incubated with secondary antibodies (Biotinylated goat anti-rabbit, Vector Labs, Burlingame, CA, United States) at room temperature for 1 h. Afterward, the slides were subjected to an ABC Substrate System (Vector Labs, Burlingame, CA) for color development and counterstained with Mayer’s hematoxylin. All IHC slides were scanned using Motic (Houston, TX) and scored by ImagePro 10 software (Vista, CA).

### Serum cytokines and chemokines

Serum inflammatory cytokines and chemokines were measured using the mouse multiplexed Luminex assays (Bio-Rad, Hercules, CA) according to the manufacturer’s protocols in the Animal Core Facility at UNC-CH. The plate was measured using the Luminex-200 system (Austin, TX).

### Statistical analysis

All experiments were repeated at least three times for consistency of results. Data were presented as mean ± SD. Statistical analysis and graphical representation were generated using GraphPad Prism eight software (La Jolla, CA). A two-sided unpaired Student’s t-test was used for comparisons between two groups with at least three replicates. One-way ANOVA with Tukey’s multiple comparison test was used when comparing multiple groups. The combination index of sulindac and PTX were calculated by CompuSym software (Biosoft, MO, United States). Statistically significant was set at P values <0.05. All *in vitro* experiments were repeated at least 3 times.

## Results

### Sulindac inhibited cell proliferation and tumor growth in OC cell lines and KpB mice

**FIGURE 1:**
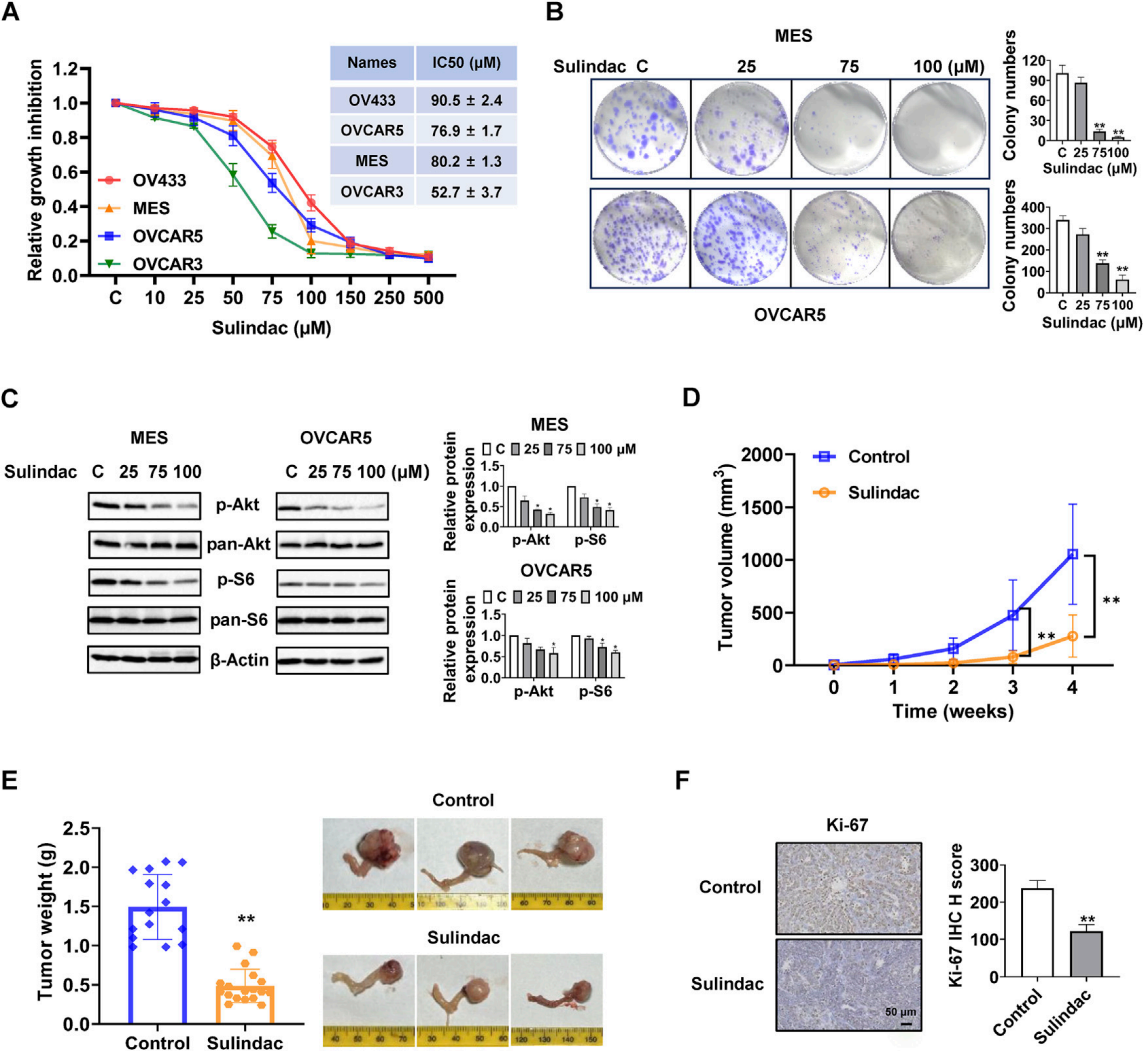
Sulindac inhibited cell proliferation and tumor growth in OC cell lines and KpB mice. MTT assay demonstrated that sulindac inhibited cell proliferation in a dose-dependent manner in OV433, OVCAR5, MES, and OVCAR3 cells after 72 h of treatment **(A)**. The MES and OVCAR5 cells were treated with sulindac for 72 h at 25, 75, and 100 μM, and then cultured for an additional 14 days. Colony assay showed that sulindac inhibited colony formation **(B)**. Sulindac reduced the expression of phosphorylated Akt and phosphorylated S6 in MES and OVCAR5 cells after 16 h of treatment **(C)**. The KpB mice were treated with sulindac (7.5 mg/kg, daily) or vehicle for 4 weeks. Sulindac effectively reduced tumor volume and tumor weight compared with control mice (n = 15) **(D,E)**. IHC results demonstrated that sulindac treatment decreased the expression of Ki-67 in OC tissues from KpB mice (n = 6) **(F)**. Data are presented as mean ± SD. Statistical significance was assessed using two-sided unpaired Student’s t-test (two groups) or one-way ANOVA with Tukey’s *post hoc* test (multiple groups). *p < 0.05, **p < 0.01, compared with control.

To investigate the effect of sulindac on cell proliferation, the OV433, MES, OVCAR5, and OVCAR3 cell lines were treated with sulindac at concentrations ranging from 10 to 500 μM for 72 h. The results of the MTT assay revealed that sulindac reduced cell proliferation in a dose-dependent manner in four OC cell lines. The mean IC50 values of sulindac were 90.5 ± 2.4 µM, 76.9 ± 1.7 µM, 80.2 ± 1.3 µM and 52.7 ± 3.7 µM for OV433, OVCAR5, MES and OVCAR3 cells, respectively (Figure 1A). Colony formation assay was used to assess the long-term effect of sulindac on cell proliferative capability. The MES and OVCAR5 cells were exposed to 25, 75, and 100 µM sulindac for 72 h and then cultured for 14 days. The results showed that 100 µM sulindac was able to reduce the colony formation of both cell lines, with the colony formation reduced by 95.0% and 81.9% in MES and OVCAR5 cells, respectively, compared to untreated cells (Figure 1B). Western blotting results demonstrated that sulindac effectively decreased the expression of phosphorylated Akt and phosphorylated S6 in both cell lines after 16 h of treatment (Figure 1C).

To investigate the effect of sulindac on tumor growth, the KpB transgenic mice of OC were treated with sulindac for 4 weeks (7.5 mg/kg, daily, oral garage) when ovarian tumors reached approximately 0.1–0.2 cm in diameter by palpation. During the treatment period, mice exhibited tolerance to sulindac treatment without any discernible changes in behavior and body weight. Sulindac-treated mice demonstrated a significant 73.7% reduction in tumor volume and 67.1% reduction in tumor weight compared with the control mice at the end of the treatment (Figures 1D,E). IHC staining showed that Ki-67 expression in sulindac-treated tumors was significantly decreased by 48.6% compared with untreated tumors (Figure 1F). These findings demonstrated sulindac is effective in inhibiting cell proliferation and tumor growth *in vitro* and *in vivo*.

### Effect of sulindac on Cox-2 pathway and chemokines in OC cells and KpB mice

**FIGURE 2:**
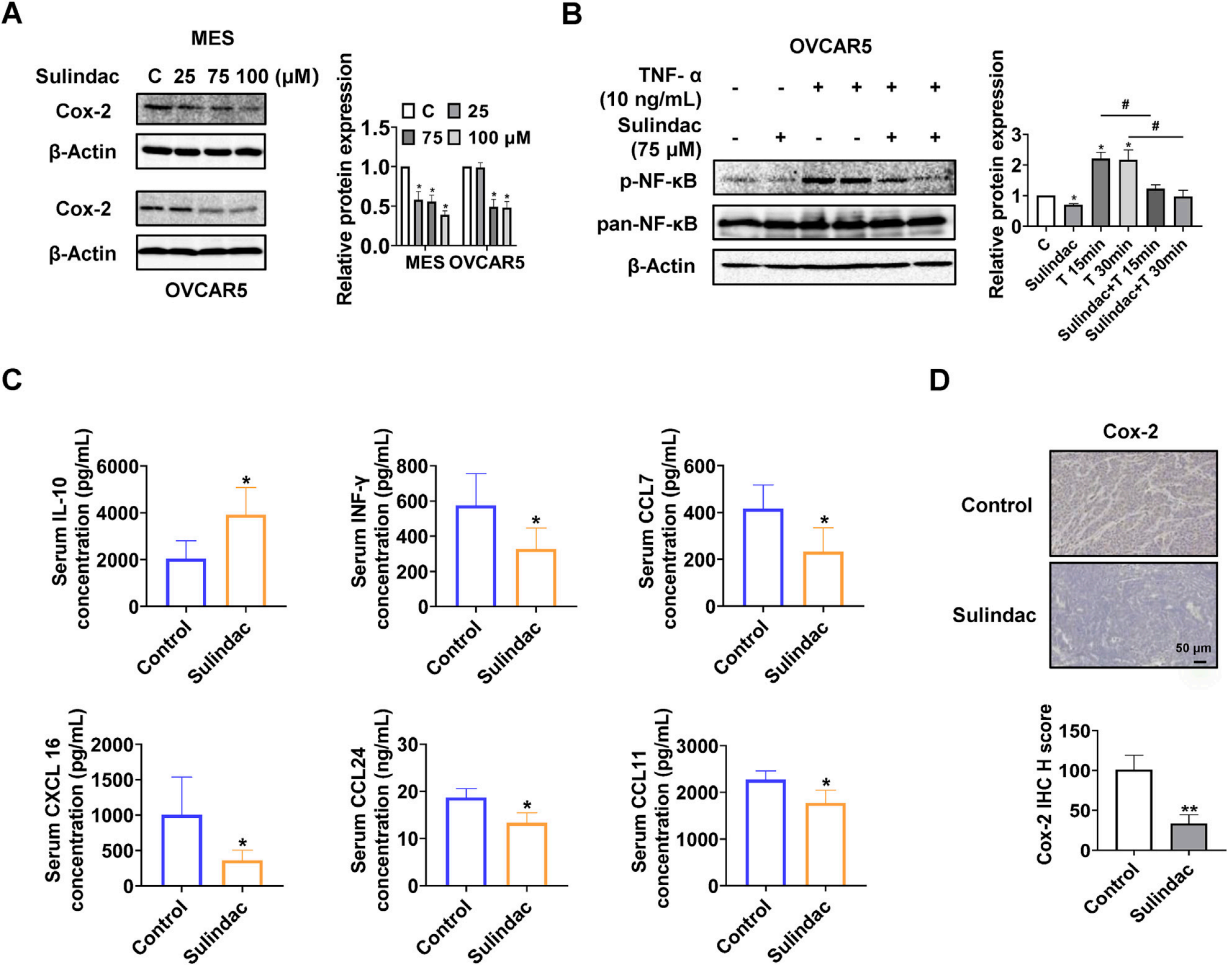
Sulindac inhibited inflammatory responses in OC cell lines and the KpB mice. Sulindac decreased the expression of Cox-2 in MES and OVCAR5 cells after 16 h of treatment **(A)**. Pretreatment of OVCAR5 cells with 75 µM sulindac for 3 h followed by treatment with 10 ng/mL TNF-α for 15 and 30 min resulted in a decrease in TNF-α-induced expression of NF-κB phosphorylation **(B)**. Sulindac treatment for 4 weeks affected serum concentrations of IL-10, INF-γ, CCL7, CXCL16, CCL24, and CCL11 in KpB mice compared with control mice (n = 15) **(C)**. IHC results showed that sulindac inhibited the expression of Cox-2 in OC tissues of KpB mice (n = 6) **(D)**. Data are presented as mean ± SD. Statistical significance was assessed using two-sided unpaired Student’s t-test (two groups) or one-way ANOVA with Tukey’s *post hoc* test (multiple groups). *p < 0.05, **p < 0.01, compared with control. #p < 0.05, ##p < 0.01, between groups.

To determine the effect of sulindac on Cox-2 pathway in OC cells, the MES and OVCAR5 cells were treated with sulindac at 25, 75, and 100 µM for 16 h. Western blotting results showed that sulindac at 75 and 100 µM significantly reduced the expression of Cox-2 in both cell lines (Figure 2A). Since NF-κB is closely involved in regulating the biological effects of sulindac and TNF-α is commonly used to induce NF-κB activity (Yi et al., 2021), OVCAR5 cells were pretreated with 75 µM sulindac for 3 h and then treated with 10 ng/mL of TNF-α for 15 and 30 min. Western blotting showed that sulindac treatment dramatically reduced the expression of phosphorylated NF-κB induced by TNF-α (Figure 2B). These results suggest that sulindac is capable of inhibiting the Cox-2 pathway and TNF-α induced NF-κB activity in OC cells.

To further investigate the effect of sulindac on inflammatory response *in vivo*, the serum levels of inflammatory cytokines and chemokines from KpB mice were measured by ELISA assay. Compared to the control group, the serum interleukin-10 (IL-10) level was significantly decreased, and the serum interferon-γ (INF-γ), chemokine (C-C motif) ligand 7 (CCL7), chemokine (C-X-C motif) ligand 16 (CXCL 16), chemokine (C-C motif) ligand 24 (CCL24), and chemokine (C-C motif) ligand 11 (CCL11) levels were decreased in the sulindac-treated group (Figure 2C). Meanwhile, the expression of Cox-2 of OC tissues in sulindac-treated mice was reduced by 66.9% compared to control mice (Figure 2D). Altogether, these results suggest that sulindac treatment for 4 weeks significantly improves the inflammatory environment *in vivo* and reduces Cox-2 activity in ovarian tumors.

### Sulindac induced cellular stress in OC cells

**FIGURE 3:**
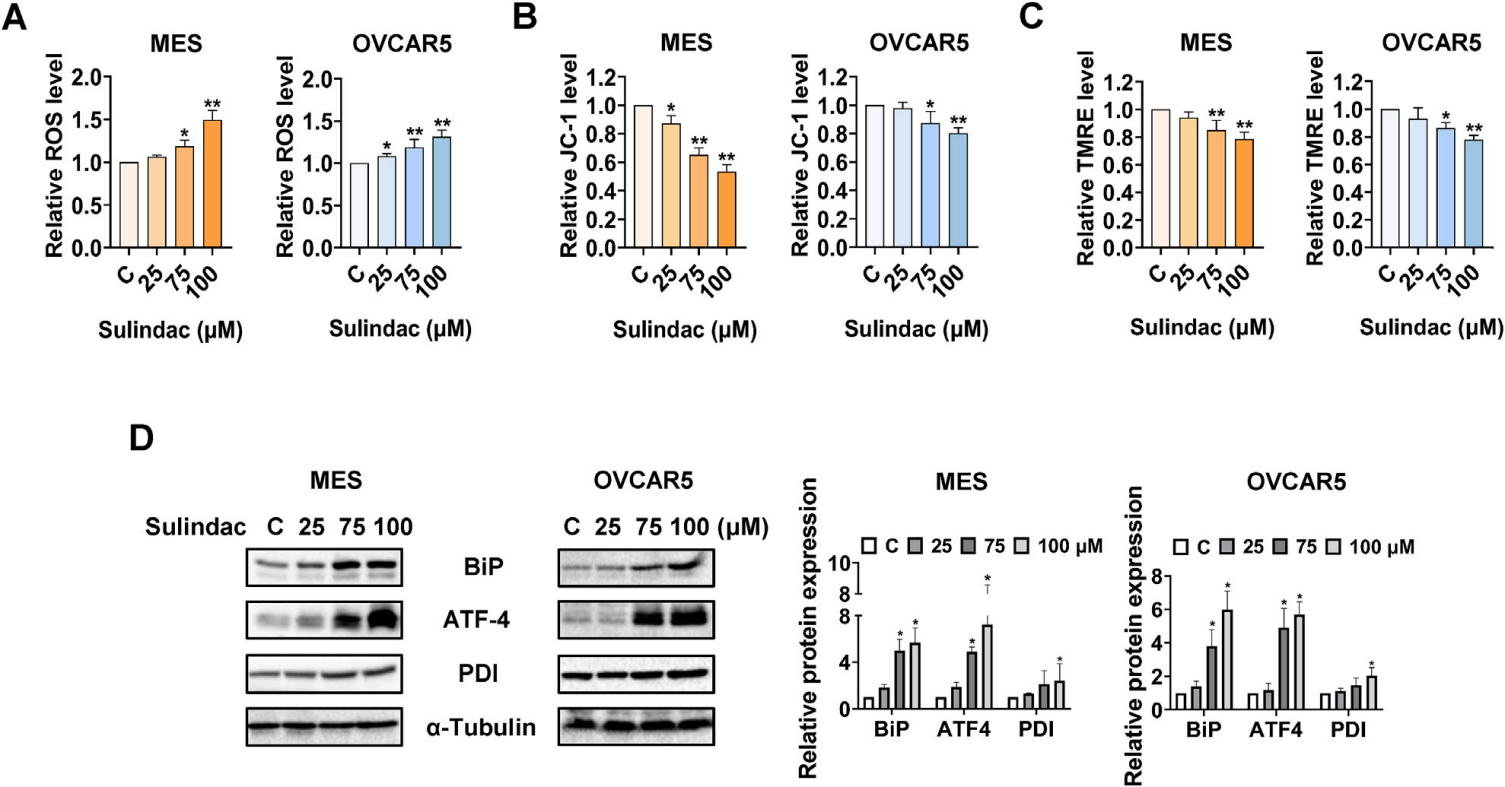
Sulindac induced cellular stress in OC cells. Sulindac significantly increased ROS levels in the MES and OVCAR5 cells after 8 h of treatment **(A)**. The JC-1 and TMRE assays demonstrated that sulindac decreased mitochondria membrane potential in the MES and OVCAR5 cells **(B,C)**. Western blotting results showed that sulindac increased expression of cell stress-related proteins such as BiP, ATF-4, and PDI in both cell lines after 8 h of treatment **(D)**. Data are presented as mean ± SD from three independent experiments. Statistical analysis was performed using one-way ANOVA with Tukey’s *post hoc* test. *p < 0.05, **p < 0.01 compared with control.

To determine the effects of sulindac on cellular stress in OC cells, the MES and OVCAR5 cells were treated with 25, 75, and 100 µM sulindac for 8 h. ROS levels were quantified using the DCFH-DA assay. Treatment with 75 and 100 μM sulindac significantly increased cellular ROS production in the MES and OVCAR5 cells. Sulindac at 100 μM increased the level of ROS by 49.3% in MES cells and 31.3% in OVCAR5 cells, respectively (Figure 3A). Next, JC-1 and TMRE assays were used to detect the effect of sulindac on mitochondrial membrane potential. Compared with control cells, sulindac at doses of 75 and 100 µM effectively reduced mitochondrial membrane potential in both cells after 6 h of treatment, with 100 µM sulindac reducing JC-1 levels by 46.7% and 20.0% in MES and OVCAR5 cells, and TMRE levels by 21.5% and 22.2% in MES and OVCAR5 cells, respectively (Figures 3B,C). Western blotting results revealed that sulindac unregulated the expression of cellular stress-related proteins BiP, ATF-4, and PDI in both MES and OVCAR5 cells following 8 h of treatment (Figure 3D). These results confirmed that sulindac exerts a pro-stress role in inhibiting cell proliferation in OC cells.

### Sulindac caused cell cycle G1 arrest in OC cells

**FIGURE 4:**
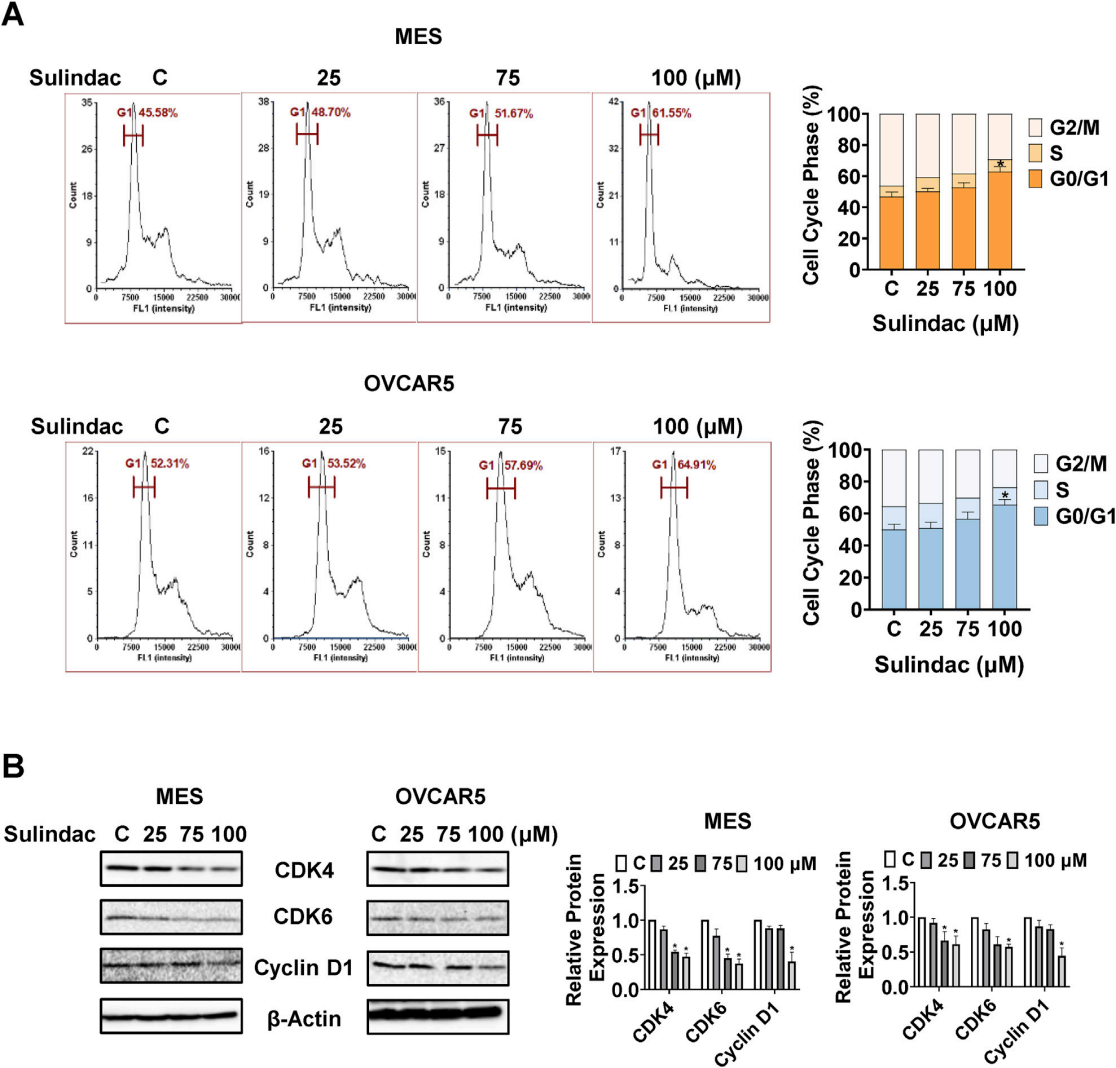
Sulindac induced cell cycle G1 arrest in OC cells. Sulindac treatment resulted in cell cycle G1 phase arrest in the MES and OVCAR5 cells after 24 h of treatment **(A)**. Western blotting results showed that sulindac inhibited the expression of CDK4, CDK6 and cyclin D1 after 24 h of treatment in both cell lines **(B)**. Data are presented as mean ± SD from three independent experiments. Statistical analysis was performed using one-way ANOVA with Tukey’s *post hoc* test. *p < 0.05, **p < 0.01 compared with control.

To investigate the potential modulation of cell cycle progression by sulindac, cell cycle profiles were measured via Cellometer after treatment of the MES and OVCAR5 cells with 25, 75 and 100 μM sulindac for 24 h 75 and 100 μM sulindac significantly induced cell cycle G1 phase arrest and decreased G2 phase in both the MES and OVCAR5 cells. Sulindac at a dose of 100 µM increased the G1 arrest phase from 46.74% in untreated cells to 62.8% in MES cells, and from 50.0% to 65.6% in OVCAR5 cells, respectively (Figure 4A). Western blotting results confirmed that after 24 h of sulindac treatment, the expression of cell cycle-related proteins such as CDK4, CDK6, and cyclin D1 were decreased in both the MES and OVCAR5 cells (Figure 4B). These data confirm that sulindac induces cell cycle G1 arrest in OC cells.

### Sulindac induced apoptosis in OC cells and ovarian tumor tissues

**FIGURE 5:**
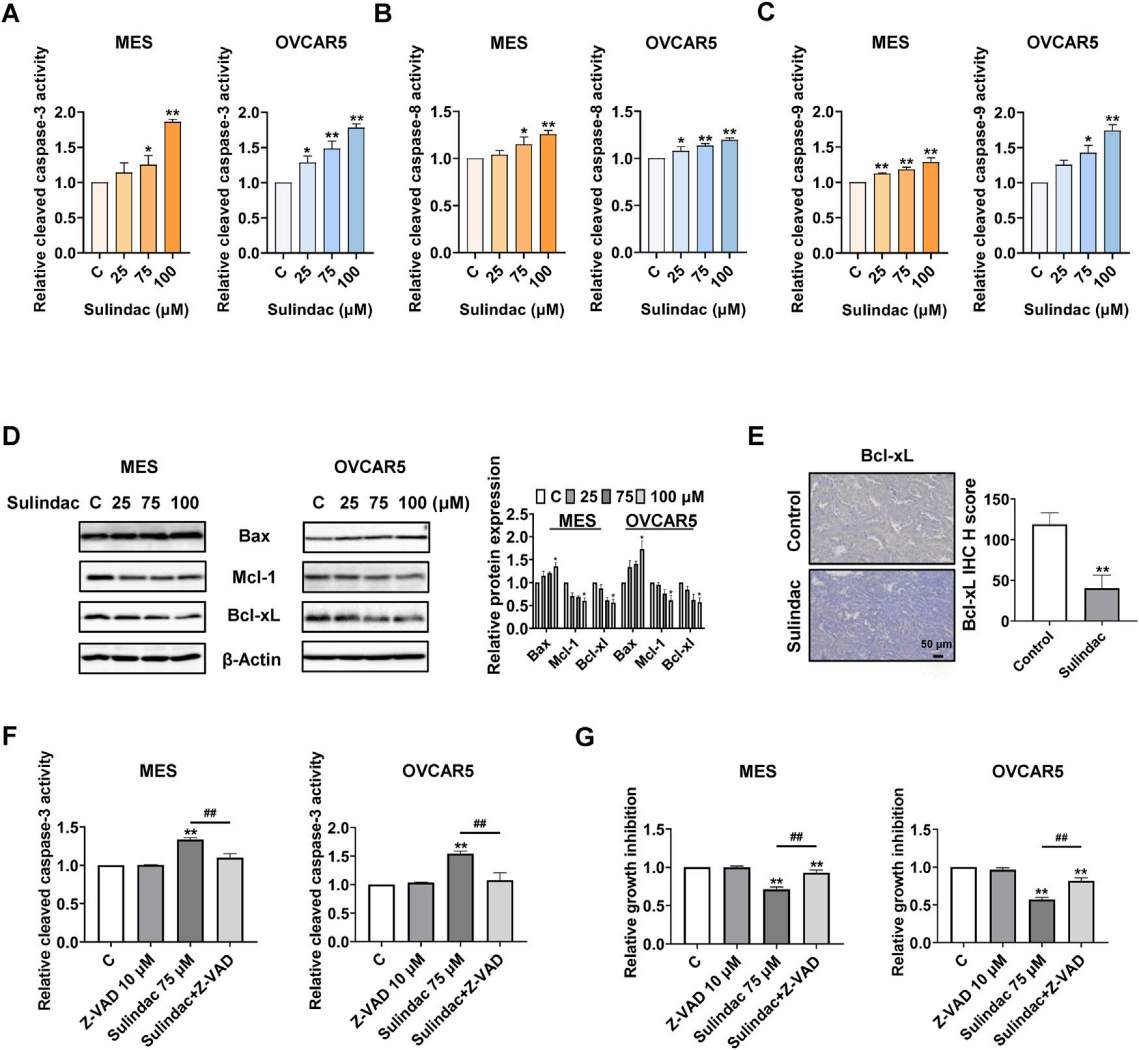
Sulindac induced apoptosis in OC cells and KpB mice. The results of ELISA assay showed that treatment with 75 and 100 µM sulindac for 14 h significantly increased the activities of cleaved caspase 3, 8, and 9 in the MES and OVCAR5 cells **(A–C)**. Sulindac increased the expression of Bax and decreased the expression of Mcl-1 and Bcl-xL in both cell lines **(D)**. IHC results revealed that sulindac inhibited the expression of Bcl-xL in OC tissues of KpB mice (n = 6) **(E)**. Z-VAD-FMK (10 µM) was pretreated with both cell lines for 2 h followed by treatment with sulindac for 14 and 72 h. Pretreatment with Z-VAD-FMK effectively blocked the 75 µM sulindac-induced cleaved caspase 3 activity **(F)**. The MTT assay showed that pretreatment with Z-VAD-FMK significantly reversed sulindac (75 µM)-induced inhibition of cell proliferation in both cell lines **(G)**. Data are presented as mean ± SD. Statistical significance was assessed using two-sided unpaired Student’s t-test (two groups) or one-way ANOVA with Tukey’s *post hoc* test (multiple groups). *p < 0.05, **p < 0.01, compared with control. #p < 0.05, ##p < 0.01, between groups.

To evaluate whether sulindac induces apoptosis in OC cells, the levels of cleaved caspase 3, 8, and 9 in the MES and OVCAR5 cells were measured by ELISA assays after 14 h of treatment. Sulindac (100 µM) significantly increased the production of cleaved caspase 3, 8, and 9 by 1.86-fold, 1.26-fold, and 1.28-fold in the MES cells, respectively, and 1.78-fold, 1.20-fold, and 1.74-fold in the OVCAR5 cells, respectively, compared with untreated cells (Figures 5A–C). Meanwhile, Western blotting showed that sulindac treatment for 8 h reduced the expression of Mcl-1 and Bcl-xl, while increasing the expression of Bax in both cell lines (Figure 5D). IHC staining further confirmed that the expression of Bcl-xL was reduced by 66.0% in ovarian tumors of KpB mice compared with the control group (Figure 5E).

To further explore the role of mitochondrial apoptotic pathway in sulindac-induced apoptosis, the MES and OVCAR5 cells were pretreated with a pan-caspase inhibitor Z-VAD-FMK (10 µM) for 2 h, followed by treatment with 75 µM sulindac. Pre-treatment with 10 μM of Z-VAD-FMK effectively blocked the increase in cleaved caspase 3 level induced by 14 h of sulindac treatment in both cell lines (Figure 5F). Similar results were observed via MTT assay. Pre-treatment with Z-VAD-FMK greatly reduced the inhibition of cell proliferation induced by 72 h of sulindac treatment in both cell lines, with inhibition decreasing from 28.9% to 7.2% in MES cells and from 43.9% to 18.3% in OVCAR5 cells, respectively (Figure 5G).

### Sulindac inhibited adhesion and invasion in OC cells

**FIGURE 6:**
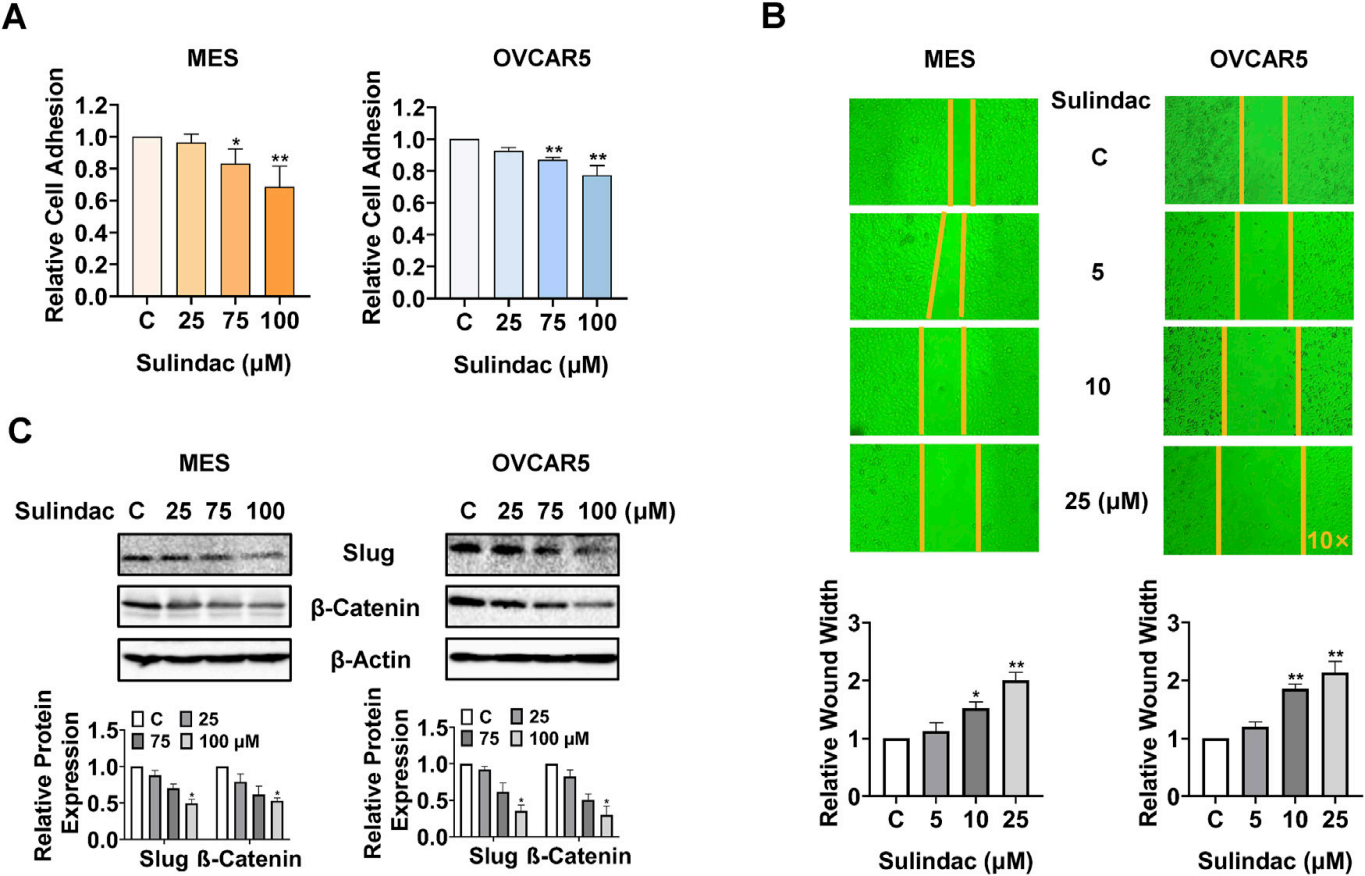
Sulindac inhibited cell adhesion and invasion in OC cells. The laminin-1 assay showed that treatment with 75 and 100 µM sulindac significantly inhibited cell adhesion in the MES and OVCAR5 cells **(A)**. The wound healing assay demonstrated that 10 and 25 µM sulindac inhibited cell migration after 24 h of treatment in the MES and OVCAR5 cells **(B)**. Western blotting showed that sulindac reduced the expression of Slug and β-Catenin after 24 h of treatment in both cell lines **(C)**. Data are presented as mean ± SD from three independent experiments. Statistical analysis was performed using one-way ANOVA with Tukey’s *post hoc* test. *p < 0.05, **p < 0.01, compared with control.

To assess the effect of sulindac on cell adhesion and migration, laminin-1 adhesion and wound healing assays were conducted in the MES and OVCAR5 cells. 75 and 100 µM sulindac significantly suppressed the cell adhesion in MES and OVCAR5 cells. Specifically, sulindac at a dose of 100 µM inhibited adhesion by 31.4% in MES cells and 22.7% in OVCAR5 cells, respectively, compared with control cells (Figure 6A). The wound healing assay demonstrated that sulindac inhibited cell migration in both cell lines. Treatment with 25 µM sulindac for 24 h significantly increased wound healing width by 2.0-fold and 2.13-fold in MES and OVCAR5 cells, respectively, compared with untreated cells (Figure 6B). Meanwhile, sulindac effectively decreased the expression of EMT-related proteins including Slug and β-Catenin after 24 h of treatment in both cells (Figure 6C). These findings indicate that sulindac has the ability to inhibit adhesion and invasion in OC cells.

### Sulindac-induced cell growth inhibition depended on cellular stress pathway

**FIGURE 7:**
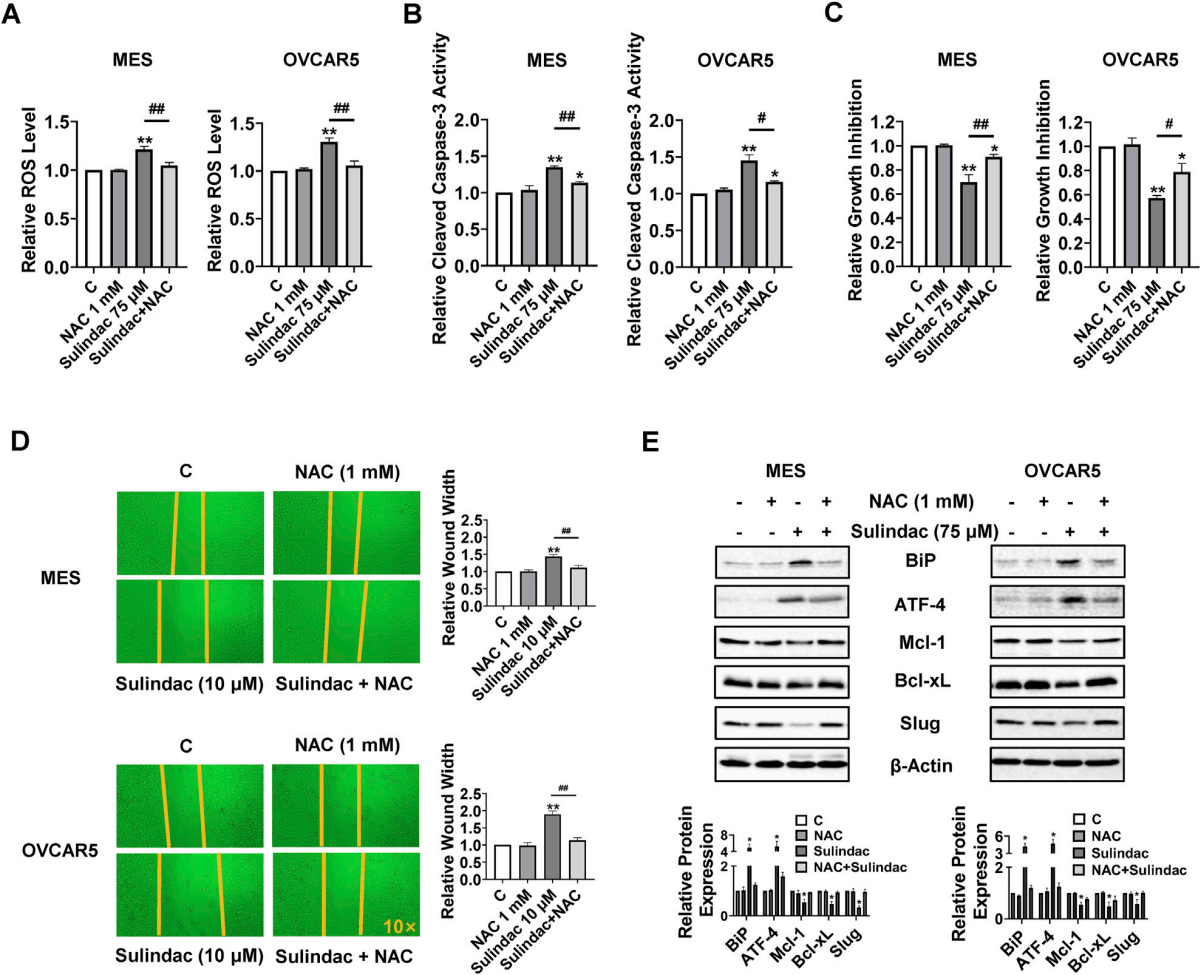
Anti-proliferative and anti-invasion activities of sulindac depends on cell stress pathway in OC cells. The MES and OVCAR5 cells were pre-treated with N-acetylcysteine (NAC, 1 mM) for 2 h and then treated with 75 µM sulindac for 8 h. ROS assays revealed that pretreatment with NAC completely reversed the sulindac-induced increase in ROS levels in both cell lines **(A)**. Pretreatment with NAC for 4 h partially reversed 75 µM sulindac-induced cleaved caspase 3 activities in the MES and OVCAR5 **(B)**. The MTT assay demonstrated that NAC partially reversed the inhibition of proliferation induced by 75 µM sulindac for 72 h in both cell lines **(C)**. Pretreatment with NAC effectively reversed the inhibition of cell migration induced by 10 µM sulindac for 24 h in the MES and OVCAR5 cells **(D)**. Western blot analysis showed that the expression of BiP, ATF-4, Mcl-1, and Bcl-xL was altered after treatment with sulindac, NAC, or the combination of sulindac and NAC **(E)**. Data are shown as mean ± SD from three independent experiments. Statistical analysis was performed using one-way ANOVA with Tukey’s *post hoc* test. *p < 0.05, **p < 0.01, compared with control. #p < 0.05, ##p < 0.01, between groups.

Given that sulindac induces cellular stress in OC cells, which is a trigger for the induction of apoptosis and cell cycle arrest, the role of cellular stress pathways in sulindac-induced cell growth inhibition was investigated. The MES and OVCAR5 cells were pretreated with the antioxidant N-acetylcysteine (1 mM) for 2 h, followed by treatment with 75 µM sulindac for 8 h. ROS assay revealed that pre-treatment with NAC totally reversed the increase in ROS levels induced by 75 µM sulindac treatment in both cell lines (Figure 7A). Pretreatment with NAC (1 mM for 4 h) also partially reversed sulindac-induced (75 µM) cleaved caspase 3 activities from 34.8% to 13.6% in MES cells and from 45.1% to 15.9% in OVCAR5 cells (Figure 7B). Similar to caspase 3 results, NAC partially reversed the inhibition of proliferation induced by 75 µM sulindac for 72 h in both cells, reducing the proliferation inhibition from 31.0% to 9.4% in MES cells and from 42.9% to 21.1% in OVCAR5 cells, respectively (Figure 7C). Importantly, blocking cell stress by NAC also partially reversed the inhibition of cell migration induced by 10 µM sulindac for 24 h, as the wound width was reduced from 40.8% to 14.4% in MES cells, and from 89.3% to 13.9% in OVCAR5 cells (Figure 7D). Western blotting confirmed that combination pretreatment with NAC and sulindac decreased the sulindac-stimulated BiP and ATF-4 expression, and increased the levels of Mcl-1, Bcl-xL, and Slug compared with monotherapy in both cell lines (Figure 7E). All these results indicate that anti-proliferative and anti-metastatic effects of sulindac are partially dependent on cellular stress pathways in OC cells.

### Sulindac synergistically enhances sensitivity to paclitaxel in paclitaxel resistant OC cell

**FIGURE 8:**
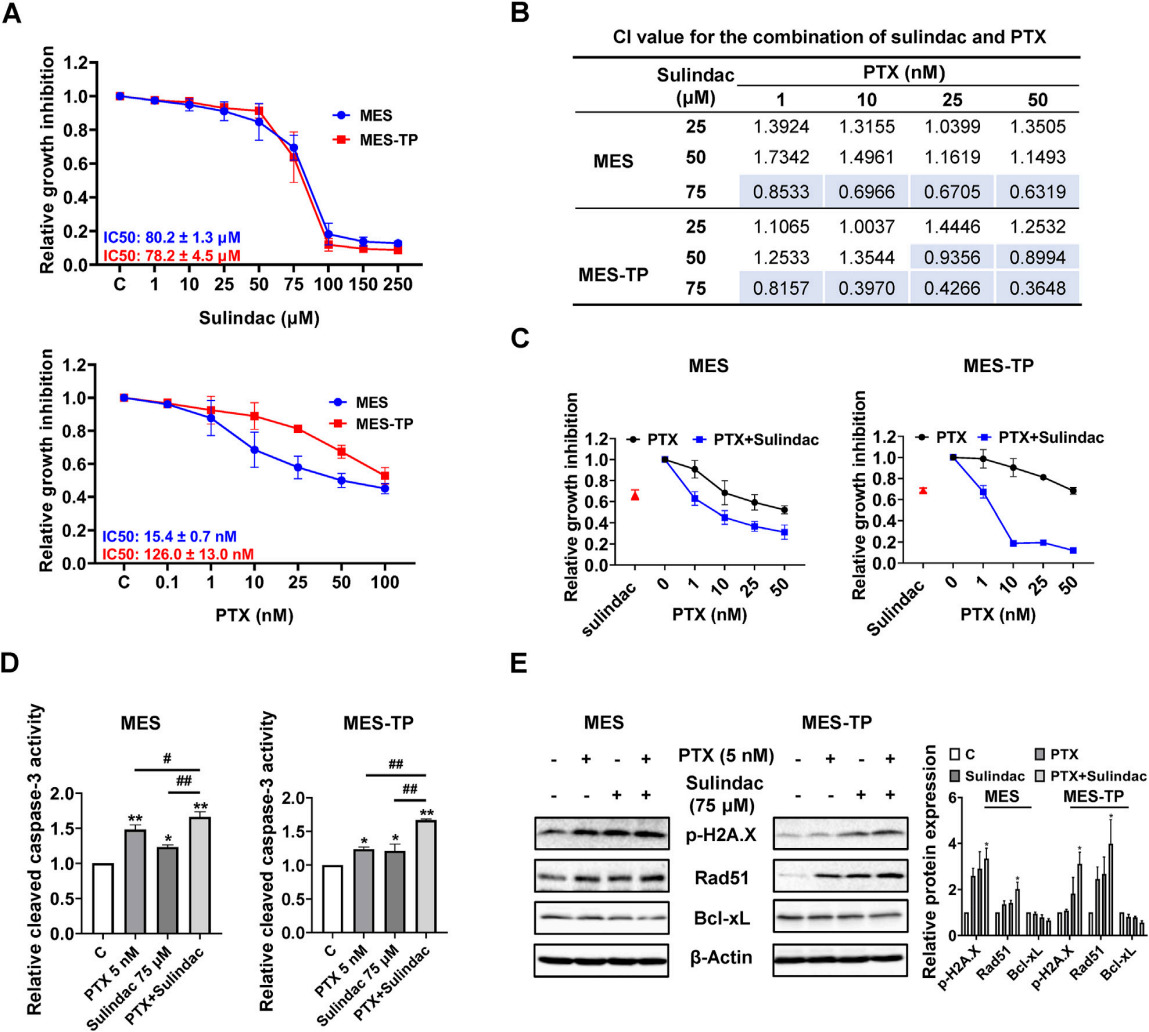
The combination of sulindac and PTX synergistically enhances cell growth inhibition in PTX-sensitive and -resistant OC cells The MES and MES-TP cells were treated with sulindac (1, 10, 25, 50, 75, 100, 150 and 250 µM), PTX (0.1, 1, 10, 25, 50 and 100 nM), and the combination for 72 h. MTT assay revealed the inhibitory effects of sulindac and PTX on cell proliferation **(A)**. The CI value for the combination of sulindac (25, 50, and 75 µM) and PTX (1, 10, 25 and 50 µM) was calculated by CompuSyn for each combination group **(B)**. MTT assay revealed changes in cell proliferation after treatment with sulindac (75 µM) and PTX (1, 10, 25, and 50 nM) in both cells **(C)**. In both cell lines, the combination of sulindac (75 µM) and PTX (5 nM) significantly increased cleaved caspace3 levels after 14 h of treatment compared with either agent alone **(D)**. Western blotting demonstrated the changes in the expression of phosphorylated H2A.X, Rad51, and Bcl-xL after treatment with sulindac, PTX or their combination in both cell lines **(E)**. Data are presented as mean ± SD from three independent experiments. Statistical analysis was performed using one-way ANOVA with Tukey’s *post hoc* test. *p < 0.05, **p < 0.01, compared with control. #p < 0.05, ##p < 0.01, between groups.

In order to explore whether sulindac and paclitaxel have synergistic effects in OC cells, the effects of sulindac combined with PTX on the cell proliferation of MES and its PTX-resistant cell line MES-TP were studied. After treatment of the two cell lines with of PTX (0.1, 1, 10, 25, 50 and 100 nM) and sulindac (1, 10, 25, 50, 75, 100, 150 and 250 µM) for 72 h, the IC50 values of PTX were different in both cell lines, with an IC50 value of 15.4 ± 0.7 nM in MES cells and an IC50 value of 126.0 ± 13.0 μM in MES-TP cells. However, they showed similar inhibitory responses to sulindac treatment (Figure 8A). According to the inhibitory response of the two cell lines to sulindac and PTX, three doses of sulindac (25, 50, and 75 µM) and four doses of PTX (1, 10, 25, and 50 nM) were chosen to co-treat the MES and MES-TP cells for 72 h. The combination index (CI) values were calculated using CompuSyn Software based on the Chou-Talalay model (Figure 8B). Sulindac at a dose of 75 µM synergistically increased the sensitivity of both cell lines to PTX (Figure 8C, CI < 1). Furthermore, 75 µM sulindac combined with 5 nM PTX produced more potent in inducing cleaved caspase 3 activity compared with either agent alone in both cell lines (Figure 8D). Western blot results showed that the combination of 75 µM sulindac and 5 nM PTX produced greater effects on decreasing expression of DNA damage markers, including phosphorylated H2A.X and Rad51, and increasing expression of the apoptotic marker Bcl-xL, than sulindac or PTX alone in both cells (Figure 8E). These results indicate that sulindac significantly increases the sensitivity of PTX both in PTX sensitive or resistant OC cells; and thus, may be useful in overcoming resistance to PTX.

## Discussion

Given that peritoneal and ovulation-induced inflammation and inflammatory mediators increase the risk of OC and promote the progression of OC, targeting inflammatory pathways has become an area of great interest for developing new therapies for OC (Savant et al., 2018; Ray et al., 2023). In this study, we explored the effect of sulindac on cell proliferation and tumor growth in human OC cell lines and the KpB transgenic mouse model of OC. Treatment with sulindac significantly inhibited cell proliferation, induced cellular stress, caused cell cycle G1 arrest and apoptosis, and suppressed cell invasion. After 4 weeks of treatment, sulindac treatment significantly reduced tumor growth, improved the inflammatory state in KpB mice, and decreased the expression of Ki-67, Cox-2 and Bcl-2 in OC tissues. Furthermore, sulindac, together with PTX, exerted a synergistic inhibitory effect on cell proliferation in both PTX-sensitive and -resistant OC cells.

Epidemiological evidence suggests that NSAIDs, including sulindac, which are associated with a reduced cancer risk, may be beneficial in patients whose tumors express PIK3CA genomic variants, as PIK3CA mutations lead to constitutive activation of the PI3K/AKT/mTOR pathway, upregulation of COX-2 activity, and increased PGE2 production (Cai et al., 2020). In our four OC cell lines and KpB mice, PIK3CA was wild-type, but p53 and KRAS status varied, suggesting that p53 and KRAS status should be considered molecular markers associated with sulindac sensitivity in OC (Song et al., 2007; Arber et al., 1997; Lawson et al., 2000). In addition to its primary targets Cox-1 and Cox-2, sulindac is thought to exert its anti-inflammatory and anti-tumor effects through other cell signaling pathways, including Wnt/β-catenin, ERK1/2 and PKG and NF-κB signaling pathways (Poursoltani et al., 2021; Rice et al., 2006; Li et al., 2013; Li et al., 2012). The functional interaction between NF-κB and COX-2 appears to be key to controlling the inflammatory process and tumor growth (Chakraborty et al., 2020; Razak et al., 2022). However, several studies have also found that sulindac demonstrated its anti-tumorigenic activity through inhibition of NF-κB activation and targeting Cox-independent pathways in cancer cells (Li et al., 2012; Lee et al., 2008; Piazza et al., 2009; Sinha et al., 2022). In OC, NF-κB and COX-2 pathways are core pathways involved in the anti-inflammatory and anti-tumor activities of NSAIDs, including sulindac, which can effectively reduce the transcription of growth factors, chemokines, and proteases that are elevated in OC (Altinoz and Korkmaz, 2004). Our results showed that sulindac can inhibit TNFα-induced NF-κB activation in OVCAR5 cells and reduced the expression of Cox-2 in both OC cell lines. In KpB mice, sulindac treatment for 4 weeks significantly reduced the Cox-2 expression in OC tissues and decreased serum inflammatory cytokines. These results imply that sulindac effectively improves the inflammatory environment *in vitro and in vivo* and inhibition of tumor cell growth by sulindac may be associated with Cox-2 dependent processes in OC. Further studies are needed to determine whether changes in NF-κB are dependent on the regulation of the Cox-2/PGE2 pathway in OC.

Previous studies have shown that sulindac increases ROS production, which is the basis for its reduction of mitochondrial membrane potential, induction of cell cycle arrest, and promotion of cancer cell apoptosis in cancer cells (Thi Thanh Nguyen and Yoon, 2024; Hwang et al., 2013; Sui et al., 2018). In agreement with these results, our results showed that sulindac treatment effectively induced ROS generation, caused mitochondrial dysfunction, and increased the expression of PDI, BiP and ATF-4 in OC cells. BiP is a direct ER stress sensor that leads to unfolded protein response (UPR) activation. Activation of PDI by increasing ROS generation can effectively lead to upregulation of PERK, thereby activating CHOP and causing functional changes in its downstream anti-apoptotic and pro-apoptotic genes, including Mcl-1, Bcl-xL and Bax, ultimately inducing cell apoptosis (Thi Thanh Nguyen and Yoon, 2024; Kranz et al., 2017). We further observed that sulindac treatment upregulated Bax expression and downregulated the expression of Mcl-1 and Bcl-xL, as well as the activation of caspases 3, 8, and 9 in OC cells, confirming that sulindac is an anti-proliferative agent that can induce the mitochondrial caspase pathway. Pretreatment with ROS scavenger NAC or pan caspase inhibitor Z-VAD-FMK produced antagonizing effects in sulindac-induced cell proliferation inhibition and apoptosis, supporting the notion that sulindac-induced ROS generation and ER stress functionally trigger apoptosis and produce cell proliferation inhibition in OC cells.

Induction of cell cycle arrest is currently believed to be one of the mechanisms by which sulindac exerts its anti-tumor activity in cancer. Sulindac can induce cell cycle G1 or G2 arrest by modulating selective cyclin/CDK complexes such as cyclin D1, cyclin G2, cyclin E, p21 and RB, as well as AKT/mTOR cell signaling pathways (Zhao et al., 2021; Gurpinar et al., 2013). Sulindac significantly induced cell cycle G1 arrest and reduced cell cycle S phase in a dose dependent manner in human breast cancer MCF-7 cells (Sui et al., 2018). Although there are no published data on the effects of sulindac on the cell cycle of OC cells, our previous studies have shown that the Cox-2 inhibitor celecoxib can effectively induce G1 arrest in OC SKOV3, Hey, and IGROV1 cells after 24 h of treatment (Suri et al., 2016). In our current study, we found that sulindac treatment resulted in cell cycle G1 arrest, along with a decrease in the expression of the cell-related proteins CDK4, CDK6 and cyclin D1 in MES and OVCAR5 cells, confirming that inhibition of cell proliferation by sulindac was involved in the processes of cell cycle in OC cells.

There is increasing evidence that increased activity of the Cox-2/PGE2 pathway promotes malignant behaviors such as cell proliferation, invasion, angiogenesis, and the epithelial-mesenchymal transition (EMT) in OC cells (Ye et al., 2020; Zhang et al., 2019). Sulindac has been shown to inhibit the migration and invasion of prostate, lung, breast and colon cancer cells by inhibiting the β-Catenin, EMT, TGFβ/miR-21, SIRT1 and AKT signaling pathways (Li et al., 2012; Stein et al., 2011; Stewart et al., 2009; Cha et al., 2016; De et al., 2016; Ko et al., 2017). Our earlier studies have shown that targeting Cox-2 by celecoxib modulates EMT and reduces angiogenesis in OC cells and KpB mice, resulting in significant reductions in cell adhesion and invasiveness (Suri et al., 2016). Our results demonstrate that sulindac inhibited OC cell adhesion and invasion and reduced the expression of Slug and β-Catenin, suggesting that the Wnt signaling pathway and EMT were involved in the process of inhibiting the invasion by sulindac in OC cells. More importantly, we found that pretreatment of MES and OVCAR5 cells with NAC significantly reversed cell migration inhibition and restored the expression of Slug in OC cells, indicating that cellular stress pathways are the triggers that control the effects of sulindac on invasion and EMT in OC cells.

Acquired paclitaxel resistance is a major cause of chemotherapy failure and poor prognosis in OC patients. Sulindac appeared to enhance the cytotoxicity of arsenic trioxide and bortezomib in colon and lung cancer cells, which was dependent on the activation of ROS generation and oxidative DNA damage (Park et al., 2008; Minami et al., 2005). Combination treatment of sulindac and docetaxel significantly increased the cytotoxicity of cisplatin-sensitive ovarian cancer cells (Barnes et al., 2007). Importantly, sulindac increased the sensitivity of anthracyclines regardless of the multidrug resistance (MDR) status in lung cancer cells (Duffy et al., 1998). In this study, we used MES and its PTX-resistant counterpart MES-TP cell line to determine the synergistic effect of sulindac and PTX on cell proliferation, apoptosis and DNA damage. Sulindac produced similar inhibitory effects and similar synergistic effects on cell growth in PTX-sensitive and resistive cell lines, even though the MDR expression level of MES-TP cells was higher than that of MES cells (data not shown). Meanwhile, the combined treatment resulted in increased cleaved caspase 3 activity, decreased Bcl-xL expression, and increased RAD51 and H2A.X expression in both cell lines compared with treatment with PTX or sulindac alone. These results support the notion that sulindac increases PTX sensitivity by increasing apoptotic activity and DNA damage, regardless of MDR activity in OC cells (Duffy et al., 1998).

Although the current study confirmed that sulindac exhibited cytotoxic effects on cell proliferation and tumor growth in OC, our study still has some limitations, such as the lack of *in vivo* data on the sulindac-paclitaxel combination and the lack of experiments exploring the effects of sulindac on normal ovarian epithelial cells to evaluate toxicity. Several studies have found that sulindac inhibits the growth of normal lung fibroblasts, normal human prostate epithelial cells, and normal mouse fibroblasts, but it has minimal cytotoxic effects compared to the same type of cancer cells and cannot even induce apoptosis in normal human prostate epithelial cells (Lim et al., 1999; Oida et al., 2005; Flis et al., 2006). In patients with APC, treatment with the sulindac derivative exisulind significantly increased apoptosis in regressing adenomas but not in normal mucosa (Lim et al., 1999). Considering the side effects of sulindac in anti-inflammatory, these results suggest that sulindac appears to be safe for cancer patients. Given that the cytotoxic effects and mechanisms of sulindac depend on the cell type and treatment concentration (Oida et al., 2005; Kung et al., 2014; Liggett et al., 2014), it is worthwhile to further investigate the effects of sulindac on normal ovarian epithelial or stromal cell growth and to evaluate the synergistic effects of sulindac combined with PTX in KpB OC model or human xenograft models of OC.

## Conclusion

This study demonstrated that sulindac has anti-proliferative, anti-inflammatory and anti-invasive effects and synergistically enhances PTX inhibition in OC cells. In addition, sulindac effectively inhibited tumor growth and improved the inflammatory environment in the KpB mice. Sulindac-induced cellular stress is the initial key step in inducing the anti-tumorigenic effects of sulindac in OC. These findings provide important biological insights for developing new clinical trials of sulindac alone or in combination with PTX in patients with OC.

## Data Availability

The raw data supporting the conclusions of this article will be made available by the authors, without undue reservation.
