# Supplementary material for: Sulindac exhibits anti-proliferative and anti-invasive effects and enhances the sensitivity to paclitaxel in ovarian cancer
Source: Front Pharmacol. 2025 Apr 30;16:1520771. doi: 10.3389/fphar.2025.1520771 (PMC12075207; doi:10.3389/fphar.2025.1520771)
Supplement: Supplementary file 1 [file DataSheet2.pdf]

Supplementary Table 1. Information on primary antibodies used for Western blotting

Antibody	Catalog Number	Target	Dilution
p-Akt	#4060	Anti-p-Akt (Ser473)	1:1000
Pan-Akt	#4691	Anti-Akt	1:1000
p-S6	#4858	Phospho-p-S6 (Ser235/236)	1:1000
Pan-S6	#2217	Anti-S6	1:1000
Cox-2	#12282	Anti-Cox-2	1:1000
p-NF-κB	#3033	Anti-p-NF-κB p65 (Ser536)	1:1000
Pan-NF-κB	#69994	Anti-pan-NF-κB p65	1:1000
BiP	#3177	Anti-BiP	1:1000
ATF-4	#11815	Anti-ATF-4	1:1000
PDI	#3501	Anti-PDI	1:1000
CDK4	#12790	Anti-CDK4	1:1000
CDK6	#13331	Anti-CDK6	1:1000
Cyclin D1	#55506	Anti-Cyclin D1	1:1000
Bax	#5023	Anti-Bax	1:2000
Mcl-1	#5453	Anti- Mcl-1	1:1000
Bcl-xL	#2764	Anti- Bcl-xL	1:1000
Slug	#9585	Anti-Slug	1:1000
β-Catenin	#8480	Anti-β-Catenin	1:1000
p-H2A.X	#9718	Anti-P-Histone H2A.X (Ser139)	1:1000
Rad51	#8875	Anti-P- Rad51	1:1000
β-Actin	#3700	Anti-β-Actin	1:2000
α-Tubulin	#2144	Anti-α-Tubulin	1:1000
