# Supplementary figures and images for: Sulindac exhibits anti-proliferative and anti-invasive effects and enhances the sensitivity to paclitaxel in ovarian cancer
Source: Front Pharmacol. 2025 Apr 30;16:1520771. doi: 10.3389/fphar.2025.1520771 (PMC12075207; doi:10.3389/fphar.2025.1520771)

Figure1

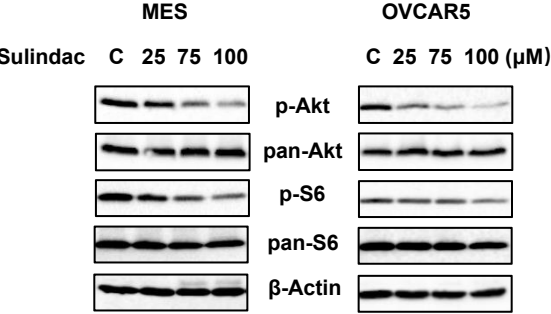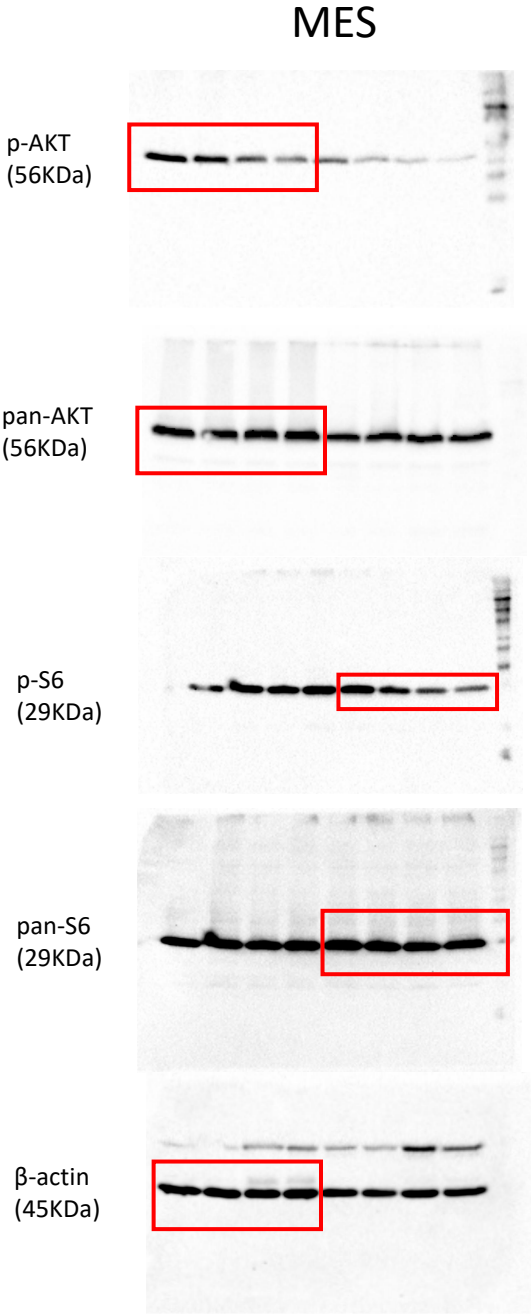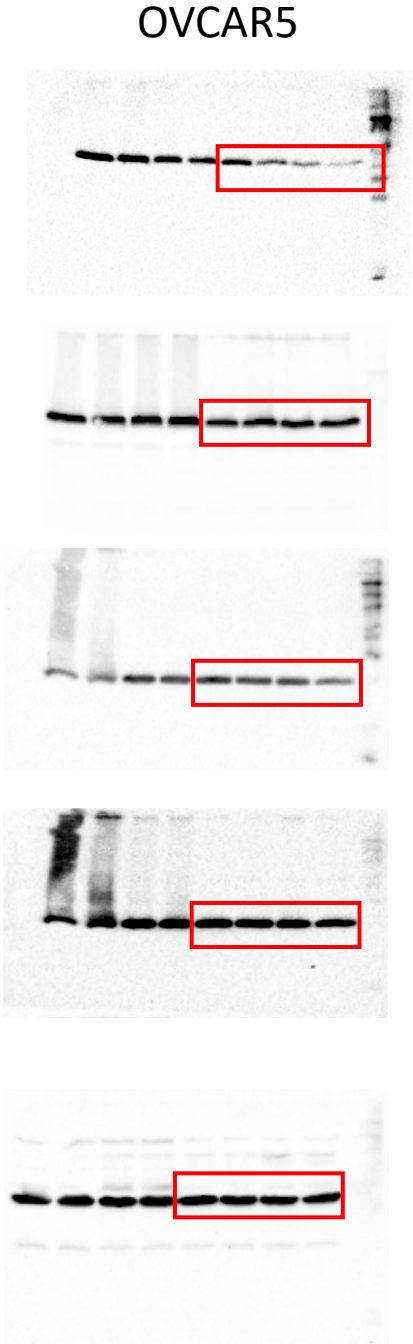

Figure2

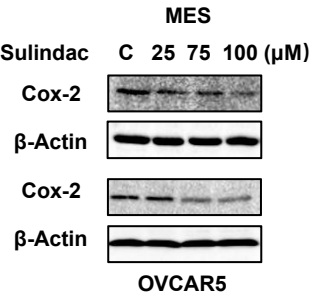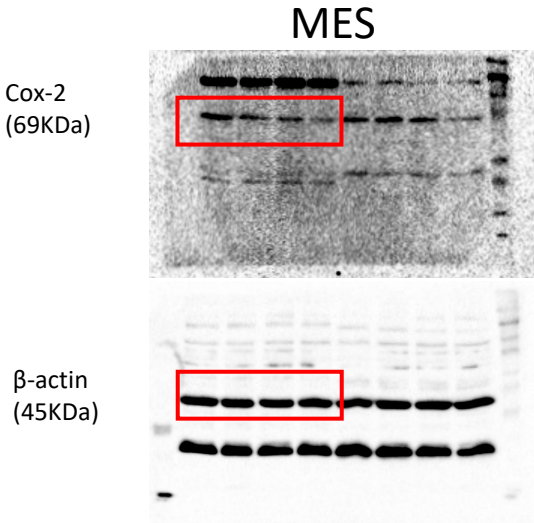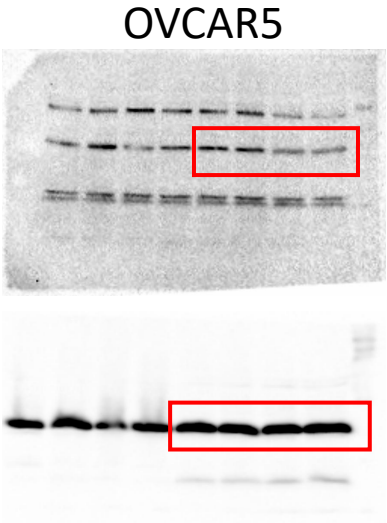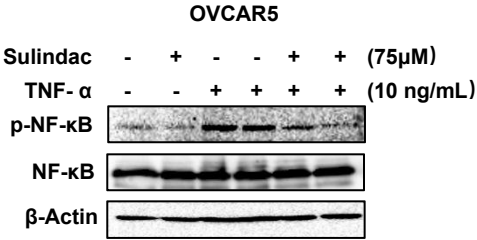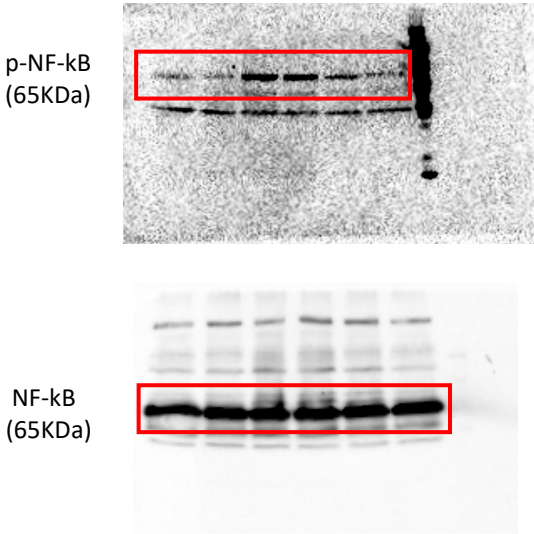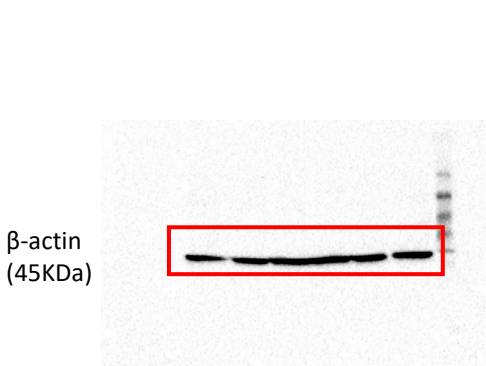

Figure3

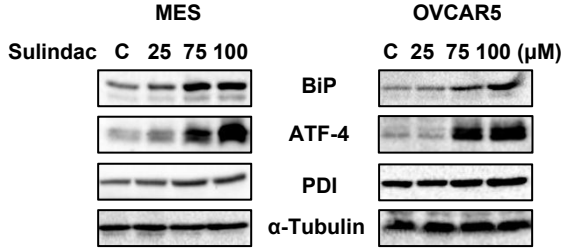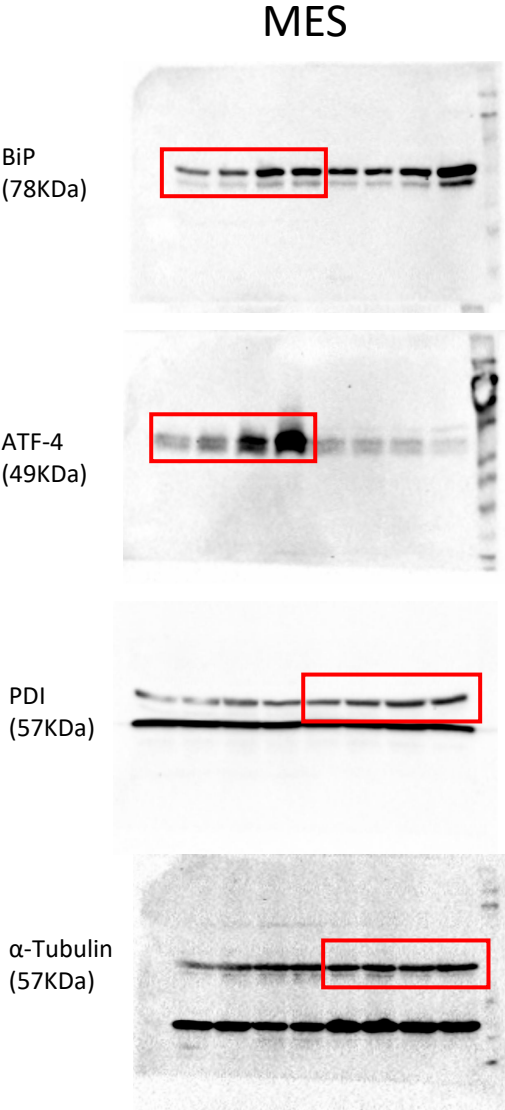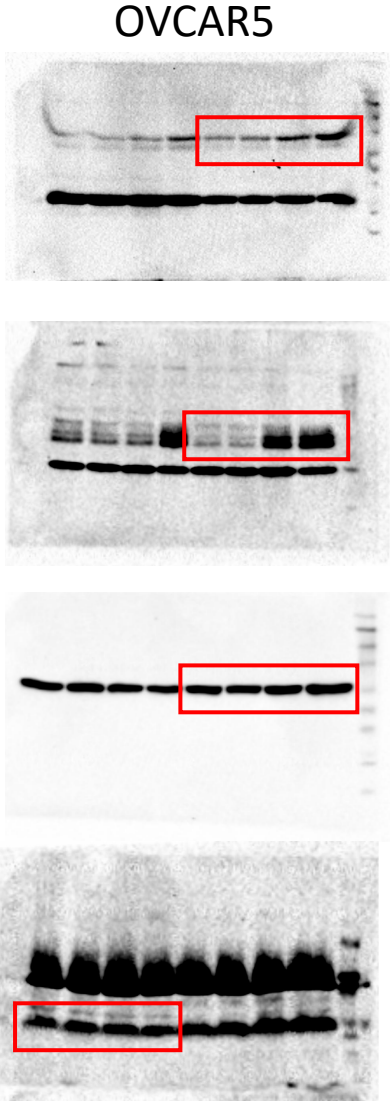

Figure4

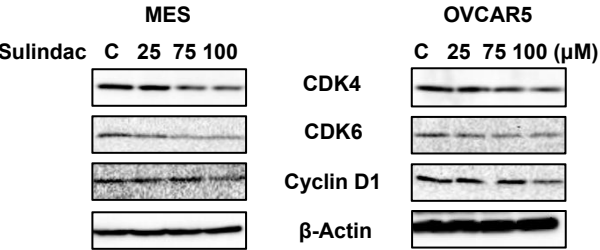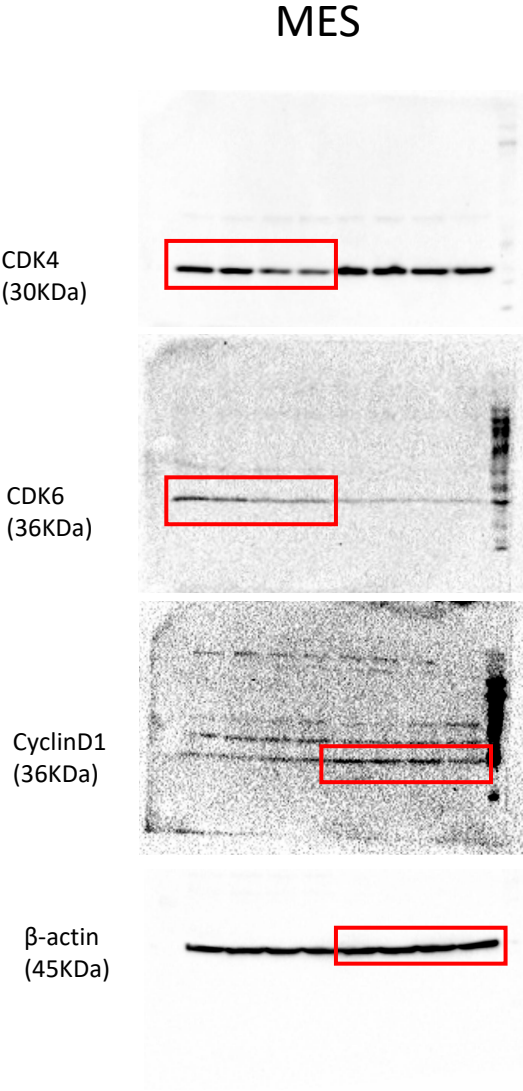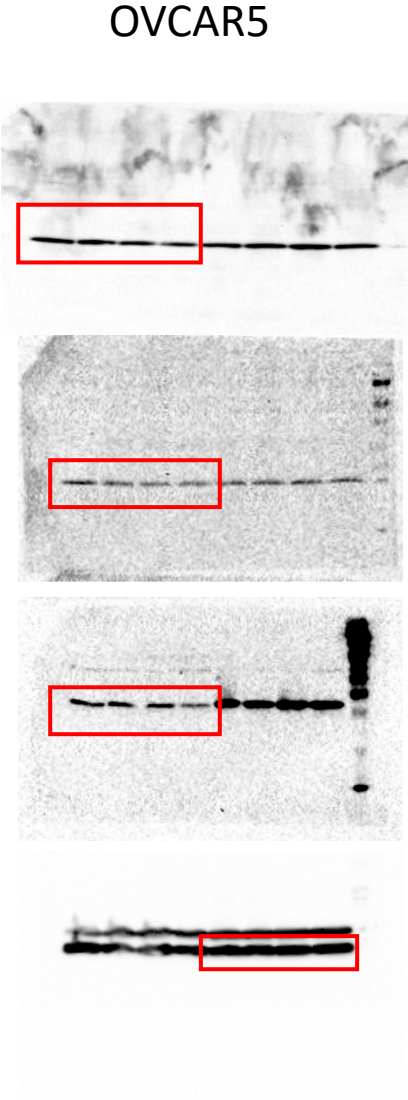

Figure5

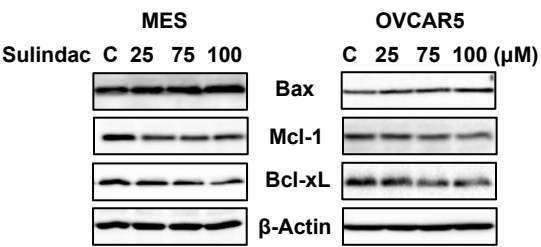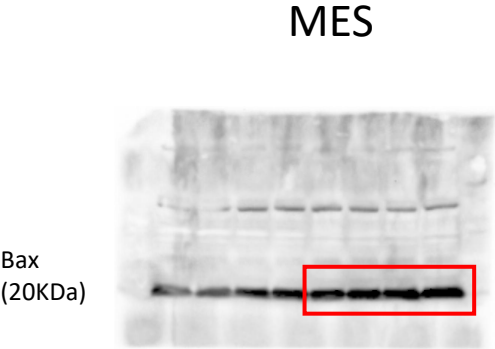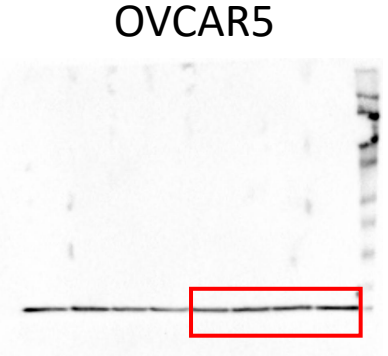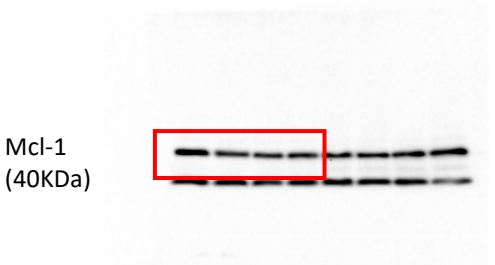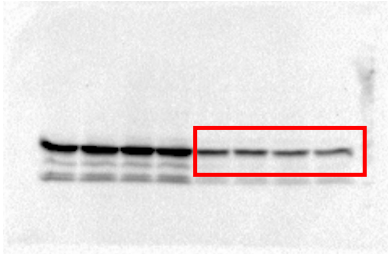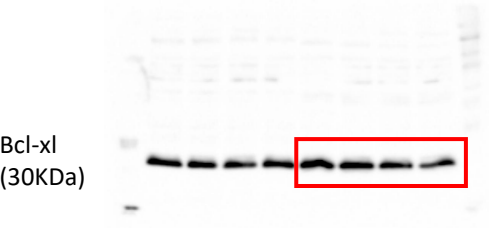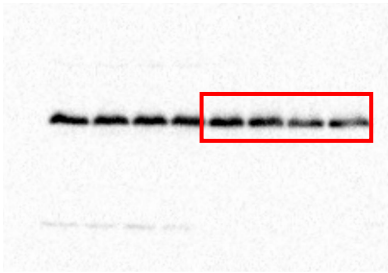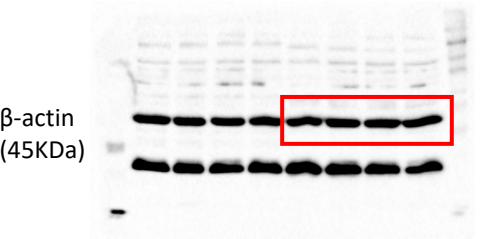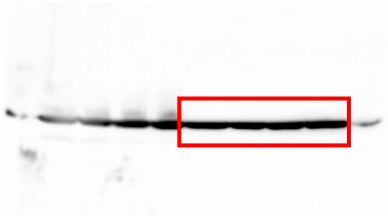

Figure6

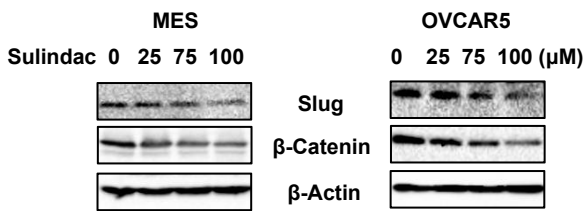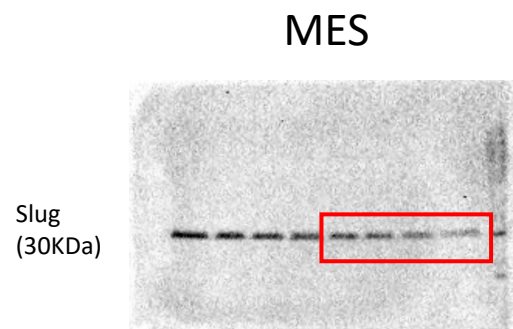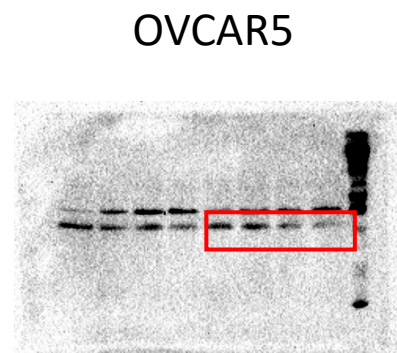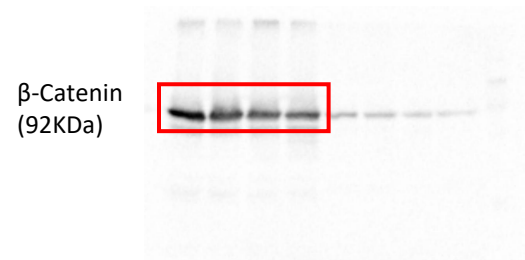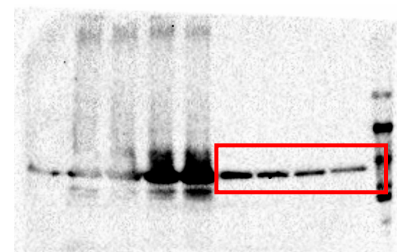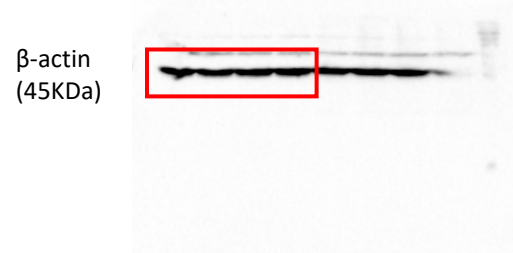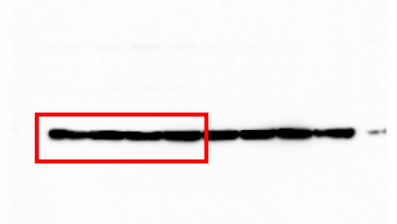

Figure7

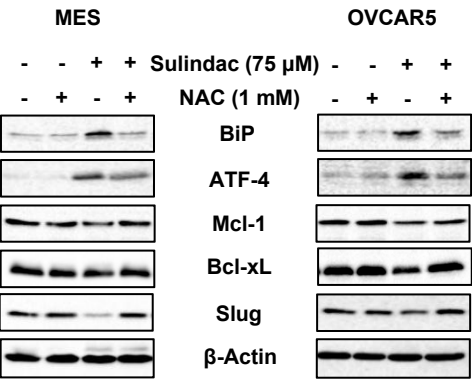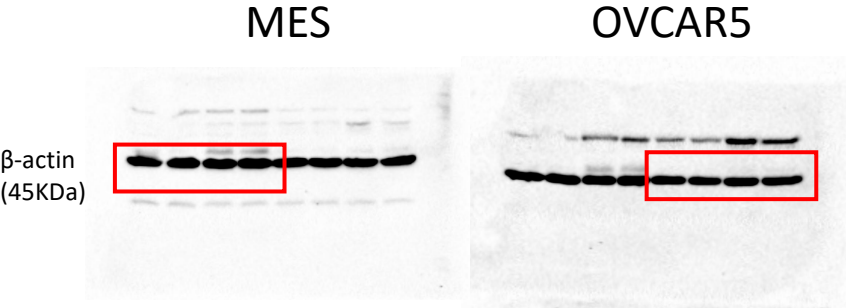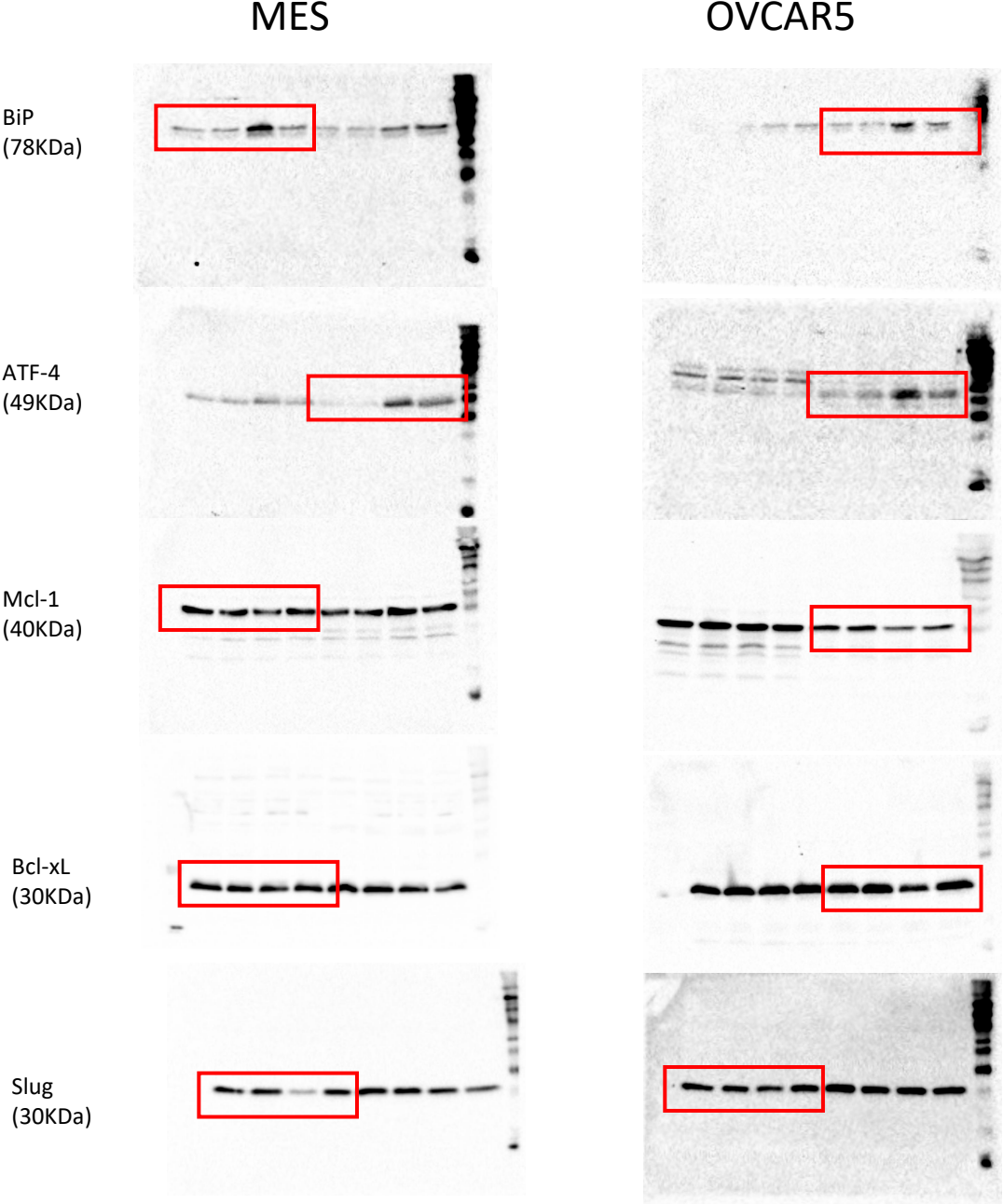

Figure8

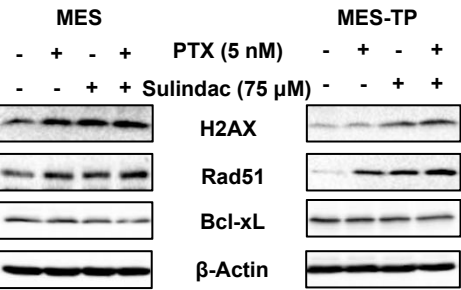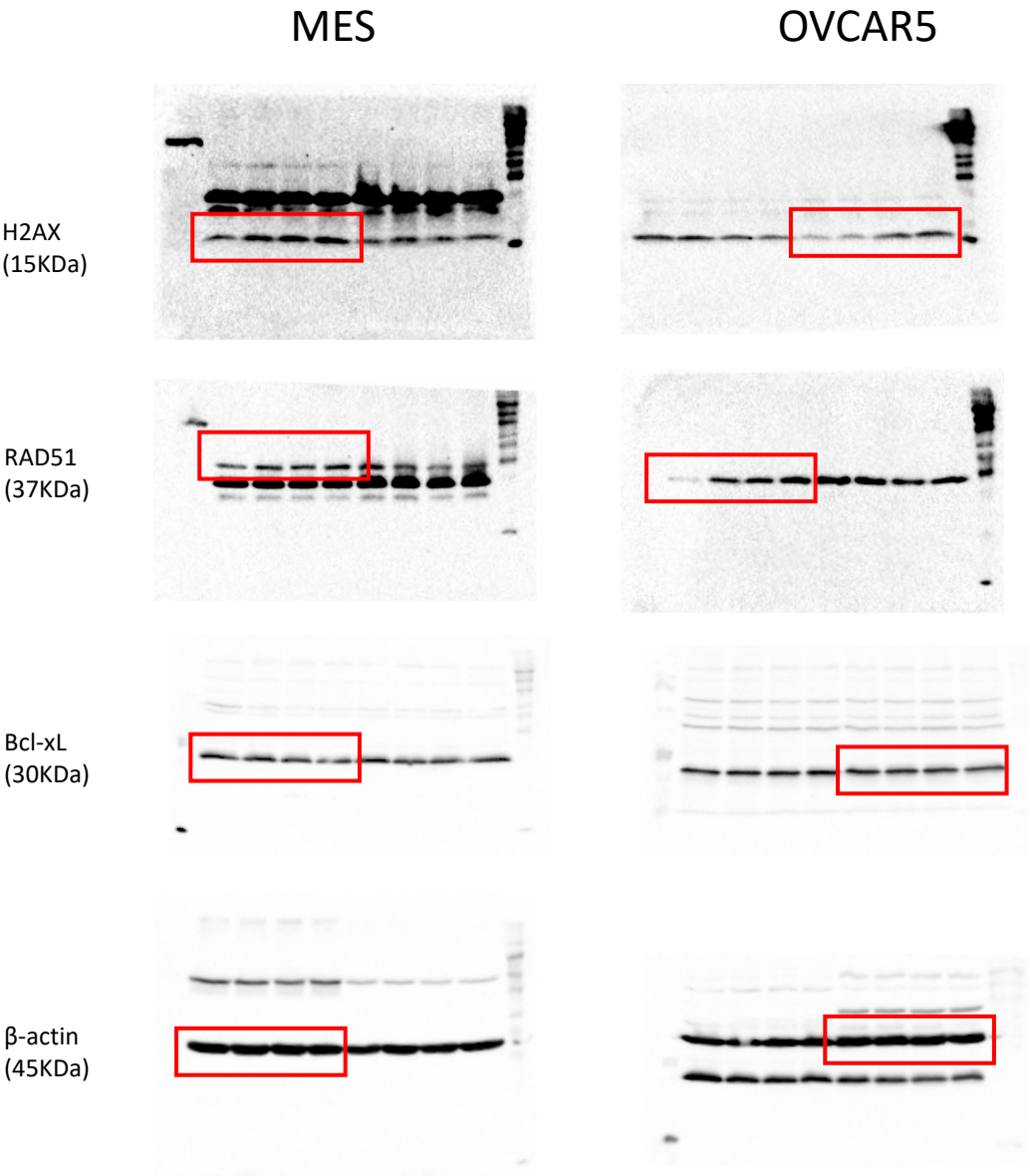

Supplement: Supplementary file 2 [file DataSheet1.pdf]
